# AABBA Graph Kernel: Atom-Atom, Bond-Bond, and Bond-Atom Autocorrelations for Machine Learning

**DOI:** 10.1021/acs.jcim.4c01583

**Published:** 2024-11-24

**Authors:** Lucía Morán-González, Jørn Eirik Betten, Hannes Kneiding, David Balcells

**Affiliations:** † Hylleraas Centre for Quantum Molecular Sciences, Department of Chemistry, University of Oslo, P.O. Box 1033 0315 Oslo, Norway; ‡ Centre for Materials Science and Nanotechnology, Department of Chemistry, University of Oslo, P.O. Box 1033 0315 Oslo, Norway; § Simula Research Laboratory, Kristian Augusts Gate 23, 0164 Oslo, Norway

## Abstract

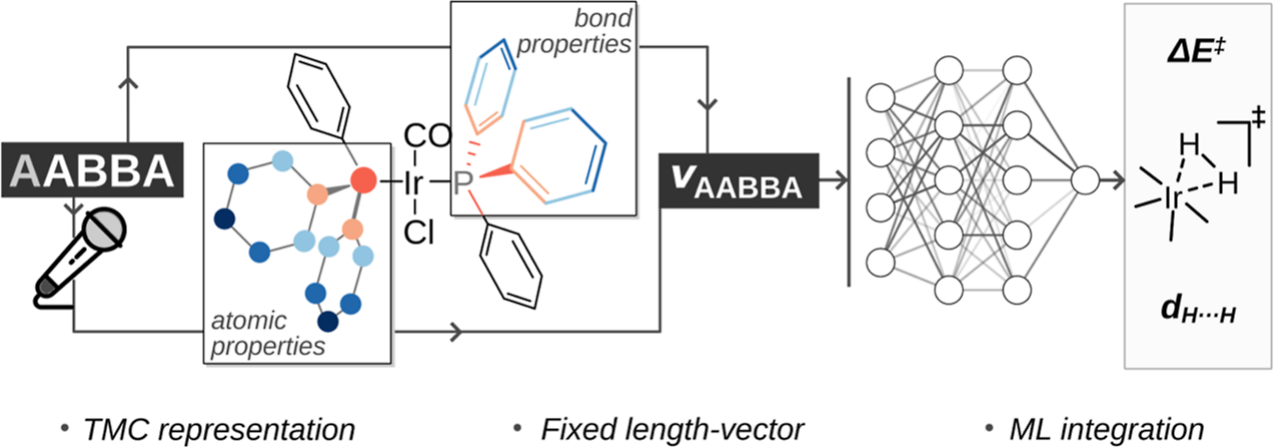

Graphs are one of the most natural and powerful representations available for molecules; natural because they have an intuitive correspondence to skeletal formulas, the language used by chemists worldwide, and powerful, because they are highly expressive both globally (molecular topology) and locally (atom and bond properties). Graph kernels are used to transform molecular graphs into fixed-length vectors, which, based on their capacity of measuring similarity, can be used as fingerprints for machine learning (ML). To date, graph kernels have mostly focused on the atomic nodes of the graph. In this work, we developed a graph kernel based on atom-atom, bond-bond, and bond-atom (AABBA) autocorrelations. The resulting vector representations were tested on regression ML tasks on a data set of transition metal complexes; a benchmark motivated by the higher complexity of these compounds relative to organic molecules. In particular, we tested different flavors of the AABBA kernel in the prediction of the energy barriers and bond distances of the Vaska’s complex data set (Friederich et al., *Chem. Sci.*, 2020, **11,** 4584). For a variety of ML models, including neural networks, gradient boosting machines, and Gaussian processes, we showed that AABBA outperforms the baseline including only atom-atom autocorrelations. Dimensionality reduction studies also showed that the bond-bond and bond-atom autocorrelations yield many of the most relevant features. We believe that the AABBA graph kernel can accelerate the exploration of large chemical spaces and inspire novel molecular representations in which both atomic and bond properties play an important role.

## Introduction

Machine learning (ML) is accelerating the fields of catalysis,^1−6^ materials science,^7−12^ and drug discovery.^13−18^ This acceleration is particularly important in the current context defined by the climate and health crises. The further development of deep,^19−21^ Bayesian,^22−24^ and ensemble^25−28^ ML methods is crucial for achieving higher levels of accuracy, generalization, and explainability. Nonetheless, research in the other key components of the ML pipeline, namely data,^29−32^ and representations,^33−35^ is also crucial, especially in the field of transition metal complex (TMC) chemistry,^36−39^ which remains incipient relative to organic chemistry. TMC data sets are smaller and more scarce, and the associated representations can fall short of capturing the intrinsic complexity of these compounds.

The representations commonly used for organic molecules are in general of limited use for TMCs. For example, line notations like SMILES^40^ are difficult to adapt to the complicated bonding patterns often observed around metal centers. More advanced notations like SELFIES^41,42^ should in principle overcome this issue but their extension toward TMCs remains unexplored. Representations based on molecular graphs are a powerful alternative, due to the higher expressivity of their topology (connectivity) and the possibility of attributing both their nodes (atoms) and edges (bonds) with geometric and electronic information,^43−46^ including NBO^47^ and QTAIM^48^ data.

**Figure 1:**
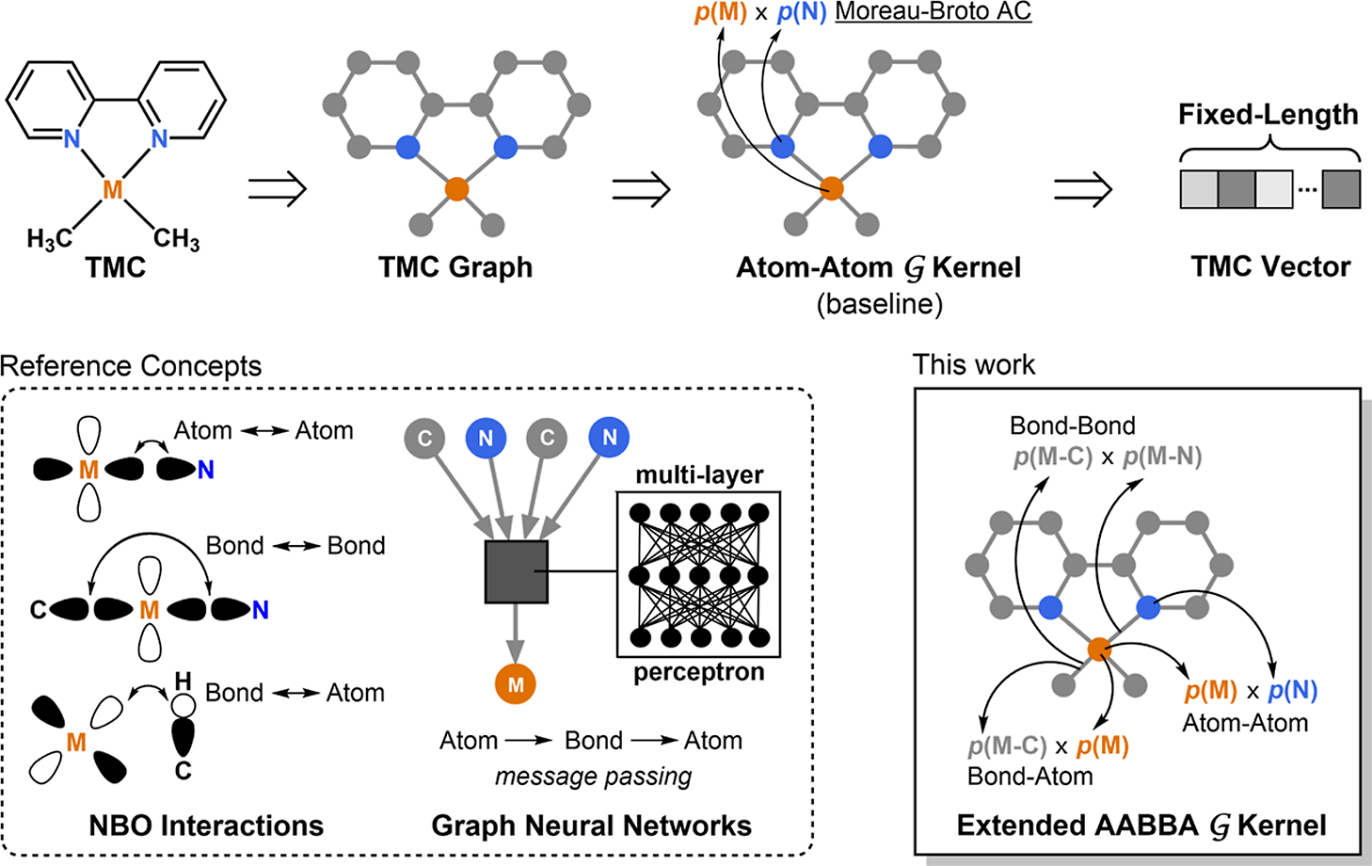
Extending the Moreau–Broto atom-atom autocorrelations to the atom-atom, bond-bond, bond-atom (AABBA) of this work. TMC = transition metal complex; AC = autocorrelation;  = graph; p = atomic and bond properties; NBO = natural bond orbital; M = Metal.

In many ML methods, graphs cannot be directly fed into the model, requiring a function, i.e. a graph kernel, to measure the similarity between graphs in a pairwise manner.^49^ In the alternative approach followed in this work, the kernel transforms graphs of any size, in a one-by-one fashion, into fixed-length vectors that, serving as molecular fingerprints, are also used to quantify similarity (Figure 1).^50^ The Moreau–Broto autocorrelation^51^ is a popular kernel of this type, consisting of an algorithm that “walks” over the molecular graph computing atomic property products (Figure 1). The algorithm iterates over the whole graph until an arbitrary depth, which is the maximum number of bonds allowed in the shortest path connecting two autocorrelated nodes. For each property, the products are added to yield the components of the final vector, which has a fixed length defined by the number of properties and the depth of the representation. Janet and Kulik adapted this approach for its application to TMCs by introducing origin and scope as autocorrelation parameters.^52−57^ Whereas the origin defines a reference node for which the property products are computed (e.g., the metal center), the scope delimits the autocorrelation to subgraph sets (e.g., axial and equatorial ligands). Further, property products were extended with additional arithmetic operations, which, for some properties, can add physical information (e.g., polarization from the subtraction of atomic electronegativities). This implementation is computationally inexpensive and it has proven its efficiency in challenging ML tasks, including the multiobjective optimization of TMCs,^5,58^ though it focuses only on the graph nodes, limiting the encoding of geometric and electronic structure information.

In this work, we introduce the atom-atom, bond-bond, bond-atom (AABBA) graph kernel, which extends the traditional Moreau–Broto atom-atom autocorrelations^51^ with bond-bond and bond-atom terms. These terms include bond properties providing both geometric (e.g., bond distance) and electronic (e.g., bond order) structure information. Figure 1 illustrates this concept for a TMC example. The idea of extracting bond-bond and bond-atom relationships with the AABBA kernel was inspired by these two theoretical frameworks; (1) natural bond orbital (NBO) analysis, in which localized atomic (i.e., lone pairs and vacancies) and bond (e.g., 2- and 3-center bonds) orbitals interact with each other;^59^ and (2) message-passing in graph neural networks, in which local chemical environments are learned by informing the atomic nodes with representations of their neighboring atoms and the bond edges connecting to them.^60,61^ The vectors generated by the AABBA kernel were leveraged in ML models predicting TMC properties, including neural networks (NNs), gradient boosting machines (GBMs), and Gaussian processes (GPs). In particular, we predicted the energy barriers and transition state bond distances of the Vaska’s complex data set. For both properties, the AABBA-based ML models achieved accuracies significantly higher than those obtained with a baseline kernel including only atom-atom terms. Further, quantitative measures of feature relevance showed that bond-bond and bond-atom terms were among the most important in the ML predictions.

## AABBA Graph Kernel

**Figure 2:**
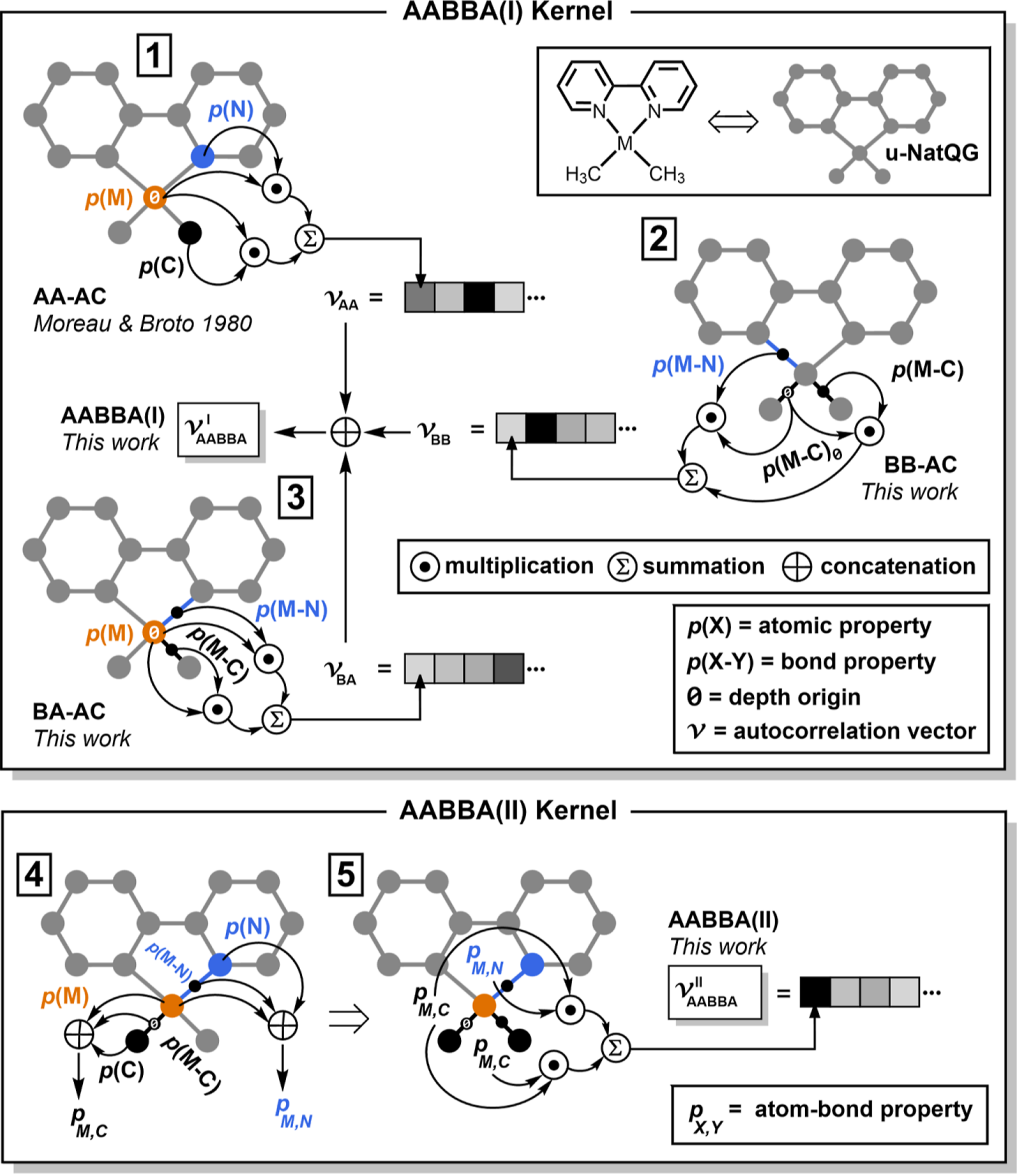
Computational graphs showing the autocorrelations of the AABBA kernels. For AABBA(I): The (1) atom-atom, (2) bond-bond, and (3) bond-atom autocorrelations. For AABBA(II): (4) Concatenation of atom and bond properties and (5) autocorrelation of the resulting atom-bond properties. For the sake of clarity, only part of the arithmetic operations are shown at depths 0 and 1. M = Metal; u-NatQG = undirected natural quantum graph.^47^

We implemented the kernel in two different flavors: AABBA(I) and AABBA(II), as illustrated in Figure 2. Both act on molecular graphs, which, for organic molecules, can be easily generated from a string representation like SMILES or the *xyz* coordinates. For TMCs, the robustness of these graph generation approaches is compromised by the complex nature of the metal–ligand bonds. This issue was addressed by using undirected natural quantum graphs (u-NatQG) derived from NBO analysis.^47^ The u-NatQG representation yielded a robust orbital-based definition of the graph topology, in which the atomic nodes and the bond edges were attributed with either generic properties (e.g., atomic number and bond order) or specific NBO electronic properties (e.g., natural atomic charges and bond orbital symmetries) computed at the PBE/def2SVP level.^62,63^

**Figure 3:**
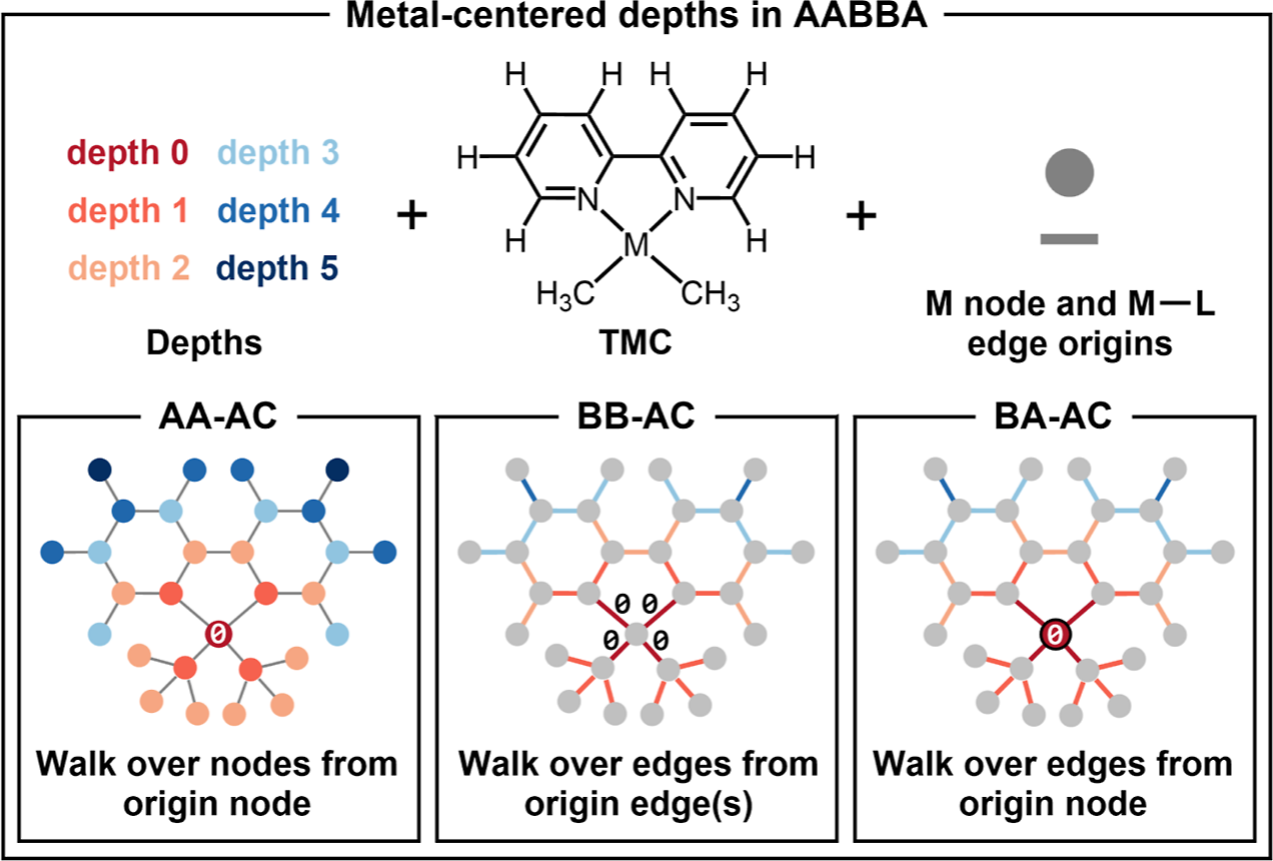
Metal-centered definition of the depth in the AABBA graph kernels. TMC = transition metal complex; M = Metal; L = ligand; AC = autocorrelation; AA = atom-atom; BB = bond-bond; BA = bond-atom; Ø = depth origin.

In the AABBA(I) kernel, the atom-atom terms are the traditional Moreau–Broto autocorrelations (AA-AC; Figure 2 and eq S1).^51^ The calculation of AA-AC can be either full (i.e., using all graph nodes as origin), or metal-centered (i.e., using only the metal node as origin). From the origin, all AA-AC terms can be extended from depth zero to an arbitrary maximum value. Figure 3 illustrates the metal-centered depth concept for all terms in the AABBA kernels. In addition to the (default) product operation, division, addition, and subtraction are also available. For the computation of the bond-bond (BB-AC; eq S16) and bond-atom (BA-AC; eqs S17–S19) terms, we implemented these two methods: (1) for the metal-centered BB-AC, we defined a “super-bond” edge origin by merging all metal–ligand bonds by either adding (BB-AC; eq S14) or averaging (-AC; eq S15) their properties and, (2) for the BA-AC, we defined arithmetic operations consistent with the dimensionality of the atomic property vectors. The final representation yielded by the AABBA(I) kernel was built by concatenating the AA-AC, -AC, and BA-AC terms (each of which can also be used separately) into a single vector (eq S25). The dimensionality of this vector could be easily augmented by expanding these terms with different properties, operators, origins, and depths.

In AABBA(II), selected atomic and bond properties associated with each atom-atom edge of the molecular graph were merged into an atom-bond property vector. For the generic properties, we defined three variants (AABBA(II)_1–3_) differing in the definition of the electronegativity and geometry components (eq S27). For the NBO properties, we defined two more variants (AABBA(II)_4–5_; eqs S28 and S29), differing in the NBO data selected for the representation. Once defined, these merged property vectors were autocorrelated following the same procedure used for BB-AC in AABBA(I); eq S16. In this case, there was no need to concatenate different terms since AABBA(II) generates the final fingerprint vector directly. The graph autocorrelated by this kernel can be seen as a junction tree in which all clusters are bonds.^64^ Further, AABBA(II) can be regarded as a dimensionality-reduced version of AABBA(I).

### Vaska’s Data Set

The Vaska’s data set^65^ used to benchmark the AABBA kernels is a curated collection of 1947 iridium complexes with diverse σ-donor, σ/π-donor, and σ-donor/π-acceptor ligands. For each complex, the data set provides computational results at the DFT(PBE/def2SVP) level^62,63^ for the transition state associated with the oxidative addition of molecular hydrogen, including the energy barrier and the breaking H···H bond distance.

### Systematic Neural Network Models

The performance of the AABBA(I) and AABBA(II) graph kernels was first assessed with the Vaska’s data set using NNs for two regression tasks, one predicting the energy barrier and the other the H···H bond distance. All models were based on a multilayer perceptron architecture with the following hyperparameters: two hidden layers with 128 nodes each, ReLU activation, Adam optimizer minimizing the mean squared error (MSE) loss, and a training/validation/test data split of 80:10:10.

The autocorrelation vectors can be computed in multiple ways depending on the property set, arithmetic operator, origin, and maximum depth. In the AABBA framework, this diversity is further expanded by the possibility of using these six graph kernels (Figure 2): AA-AC, BB-AC, -AC, BA-AC, AABBA(I), and AABBA(II). As a first approach, we built the autocorrelation vectors in a systematic manner by gradually increasing their dimensionality, based on the results obtained with each increment (Tables 1 and 2). When formulating the autocorrelations, we took into account their compared performances; i.e., some of the models that worked well or poorly for barrier prediction were tested or excluded, respectively, for distance prediction, and vice versa, also prompting the optimization of specific models for either of the two tasks; therefore, the models listed in both tables are not exactly the same.

**Table 1:** Accuracy in the Prediction of the Vaska’s Dataset Energy Barriers with Neural Networks on the Test Split^a^

model	input						average error^b^		lowest error^b^	
	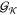	prop	Ôp	Ø^c^	*D*	dim^d^	MAE	*r*^2^	MAE	*r*^2^
**1**	**AA**	**P**^e^	**⊙**^f^	**MC**	**3**	**18**	**1.22±0.02**	**0.844±0.007**	**1.16**	**0.850**
2	BB	P	⊙	MC	3	12	1.42 ± 0.03	0.801 ± 0.005	1.37	0.803
**3**	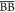	**P**	**⊙**	**MC**	**3**	**10**	**1.40±0.04**	**0.765±0.016**	**1.26**	**0.791**
4	BA	P	⊙	MC	3	20	2.14 ± 0.04	0.571 ± 0.013	2.05	0.595
**5**	**I**^a^	**P**	**⊙**	**MC**	**3**	**48**	**0.90±0.02**	**0.914±0.003**	**0.86**	**0.916**
6	I	P	⊖_χ_^f^	MC	3	47	0.91 ± 0.01	0.913 ± 0.003	0.89	0.919
7	I	P	⌀_*R*_^f^	MC	3	47	0.92 ± 0.02	0.911 ± 0.004	0.89	0.919
8	I	NBO^h^	⊙	MC	3	212	0.89 ± 0.02	0.908 ± 0.007	0.85	0.913
**9**	**I**	**NBO**	**⊙**	**F**	**3**	**220**	**0.79±0.02**	**0.927±0.002**	**0.76**	**0.928**
10	I	NBO	⊙	MC	4	263	0.90 ± 0.02	0.899 ± 0.008	0.84	0.925
11	I	NBO	⊙	MC	5	298	0.90 ± 0.02	0.904 ± 0.007	0.87	0.899
**12**	**I**	**NBO**	**⊙**	**MC**	**6**	**303**	**0.88±0.02**	**0.907±0.007**	**0.84**	**0.918**
13	I^i^	NBO	⊙	F	3	223	0.78 ± 0.02	0.928 ± 0.004	0.73	0.933
**14**	**II**_**1**_^g^	**P**	**⊙**	**MC**	**3**	**33**	**0.94±0.02**	**0.906±0.002**	**0.89**	**0.913**
15	II_2_	P	⊙	MC	3	29	0.96 ± 0.03	0.904 ± 0.005	0.86	0.917
16	II_3_	P	⊙	MC	3	33	0.94 ± 0.02	0.908 ± 0.004	0.90	0.913
17	II_4_	NBO	⊙	MC	3	92	0.86 ± 0.02	0.918 ± 0.003	0.82	0.926
18	II_5_	NBO	⊙	MC	3	80	0.94 ± 0.02	0.907 ± 0.004	0.88	0.915
19	II_4_	NBO	⊙	F	3	98	1.15 ± 0.03	0.850 ± 0.007	1.05	0.862
20	II_4_	NBO	⊙	MC	4	111	0.88 ± 0.02	0.916 ± 0.005	0.85	0.914
**21**	**II**_**4**_	**NBO**	**⊙**	**MC**	**5**	**129**	**0.85±0.03**	**0.916±0.004**	**0.81**	**0.923**

a The inputs passed to the models were vectors defined with different graph kernels , property types (prop), operators (Ôp), origins (Ø), and maximum depths (*D*), yielding different dimensionality (dim). The mean absolute error (MAE) is given in Kcal/mol.

b From ten repetitions with a training:validation:test split of 80:10:10.

c Metal-centered (MC) or full (F).

d After removing redundant dimensions.

e I.e. P_A_, P_B_, and P_AB_ periodic and generic property sets.

f All properties correlated by product (⊙), except the electronegativity in entry 6 (subtracted, ⊖_χ_), and the covalent radius in entry 7 (divided, ⌀_*R*_).

g Entries 5–13 and 14–21 correspond to the AABBA(I) and AABBA(II) kernels.

h I.e. P_A,NBO_, P_B,NBO_, and P_AB,NBO_ NBO property sets.

i Including whole-graph properties. See the Supporting Information for further details.

**Table 2:** Accuracy in the Prediction of the Vaska’s Dataset H···H Distances with Neural Networks on the Test Split^a^

model	input						average error^b^		lowest error^b^	
	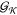	Prop	Ôp	Ø^c^	*D*	dim^d^	MAE	*r*^2^	MAE	*r*^2^
**23**	**AA**	**P**^e^	**⊙**^f^	**MC**	**3**	**18**	2.38 × **10**^**–2**^ ± 9 × 10^–4^	**0.687±0.012**	2.11 × **10**^**–2**^	**0.727**
24	BB	P	⊙	MC	3	12	2.30 × 10^–2^ ± 4 × 10^–4^	0.706 ± 0.007	2.21 × 10^–2^	0.714
**25**	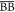	**P**	**⊙**	**MC**	**3**	**10**	2.35 × **10**^**–2**^ ± 1.0 × 10^–3^	**0.673±0.020**	2.09 × **10**^**–2**^	**0.729**
26	BA	P	⊙	MC	3	20	3.03 × 10^–2^ ± 6 × 10^–4^	0.537 ± 0.012	2.91 × 10^–2^	0.551
**27**	**I**^g^	**P**	**⊙**	**MC**	**3**	**48**	2.07 × **10**^**–2**^ ± 4 × 10^–4^	**0.747±0.006**	1.93 × **10**^**–2**^	**0.767**
28	I	P	⊙	F	3	220	2.12 × 10^–2^ ± 7 × 10^–4^	0.716 ± 0.015	1.98 × 10^–2^	0.739
29	I	P	⊙	F^h^	3	220	2.54 × 10^–2^ ± 1.0 × 10^–3^	0.632 ± 0.019	2.37 × 10^–2^	0.669
**30**	**I**	**P**	**⊖**_**χ**_^f^	**MC**	**3**	**47**	1.96 × 10^–2^ ± 5 × 10^–4^	**0.767±0.007**	1.87 ×10^–2^	**0.769**
31	I	P	⌀_*R*_^f^	MC	3	47	2.04 × 10^–2^ ± 6 × 10^–4^	0.764 ± 0.004	1.96 × 10^–2^	0.761
32	I	NBO^i^	⊙	MC	3	212	2.27 ×10^–2^ ± 8 ×10^–4^	0.663 ± 0.021	2.13 × 10^–2^	0.702
33	I	NBO	⊙	MC	4	263	2.22 × 10^–2^ ± 7 × 10^–4^	0.676 ± 0.012	2.12 × 10^–2^	0.690
34	I	NBO	⊙	MC	5	298	2.16 ×10^–2^ ± 6 ×10^–4^	0.705 ± 0.020	2.04 × 10^–2^	0.706
**35**	**I**	**NBO**	**⊙**	**F**	**5**	**316**	2.10 × 10^–2^ ± 1.0 × 10^–3^	**0.718±0.021**	1.89 × 10^–2^	**0.748**
36	I	NBO	⊙	F^h^	5	316	2.60 ×10^–2^ ± 5.2 ×10^–3^	0.586 ± 0.153	2.06 × 10^–2^	0.727
37	I	NBO	⊙	MC	6	303	2.17 × 10^–2^ ± 7 × 10^–4^	0.704 ± 0.016	1.99 × 10^–2^	0.745
38	II_1_^g^	P	⊙	MC	3	33	1.91 ×10^–2^ ± 4 ×10^–4^	0.771 ± 0.009	1.81 × 10^–2^	0.788
39	II_2_	P	⊙	MC	3	29	1.93 × 10^–2^ ± 4 × 10^–4^	0.768 ± 0.006	1.81 × 10^–2^	0.772
**40**	**II**_**3**_	**P**	**⊙**	**MC**	**3**	**33**	1.85 × 10^–2^ ± 5 × 10^–4^	**0.781±0.009**	1.72 × 10^–2^	**0.804**
41	II_3_	P	⊙	F	3	54	1.97 ×10^–2^ ± 7 ×10^–4^	0.747 ± 0.013	1.87 × 10^–2^	0.765
42	II_4_	NBO	⊙	MC	3	92	2.21 × 10^–2^ ± 5 × 10^–4^	0.685 ± 0.013	2.13 × 10^–2^	0.704
43	II_5_	NBO	⊙	MC	3	80	2.20 ×10^–2^ ± 6 ×10^–4^	0.687 ± 0.024	2.05 × 10^–2^	0.749
44	II_3_	P	⊙	MC	4	42	1.87 × 10^–2^ ± 4 × 10^–4^	0.776 ± 0.009	1.82 × 10^–2^	0.785
45	II_3_	P	⊙	MC	5	51	1.86 × 10^–2^ ± 5 × 10^–4^	0.766 ± 0.011	1.78 × 10^–2^	0.795
46	II_3_	P	⊙	MC	6	51	1.86 × 10^–2^ ± 4 × 10^–4^	0.770 ± 0.008	1.74 × 10^–2^	0.792
47	II_3_^j^	P	⊙	MC	3	36	1.86 × 10^–2^ ± 4 × 10^–4^	0.777 ± 0.008	1.74 × 10^–2^	0.799

a The inputs passed to the models were vectors defined with different graph kernels , property types (prop), operators (Ôp), origins (Ø), and maximum depths (*D*), yielding different Dimensionality (dim). The mean absolute error (MAE) is given in Å.

b From ten repetitions with a training:validation:test split of 80:10:10.

c Metal-centered (MC) or full (F).

d After removing redundant dimensions.

e I.e. P_A_, P_B_, and P_AB_ periodic and generic property sets.

f All properties correlated by product (⊙), except the electronegativity in entry 8 (subtracted, ⊖_χ_), and the covalent radius in entry 9 (divided, ⌀_R_).

g Entries 27−37 and 38−47 correspond to the AABBA(I) and AABBA(II) kernels, respectively.

h From an extended neural network of 3 hidden layers with 256 nodes each.

i I.e. P_A,NBO_, P_B,NBO_, and P_AB,NBO_ NBO property sets.

j Also including whole-graph properties. See the Supporting Information for further details.

### Energy Barrier

The results collected in Table 1 for the prediction of the oxidative addition barrier show that the Moreau–Broto AA-AC achieved a mean absolute error (MAE) of 1.16 kcal/mol, using the metal-centered autocorrelation of generic properties at a maximum depth of three (model 1). Following the same approach, we also tested the autocorrelations leveraging bond properties, i.e. the BB-, -, and BA-AC. In line with the smaller amount of properties describing the bonds (three) relative to the atoms (nine), these autocorrelations showed a poorer performance, though -AC achieved a remarkable accuracy, with MAE = 1.26 kcal/mol (model 3). Further, when the associated autocorrelation vector was concatenated with those extracted by the AA- and BA-AC kernels, the resulting AABBA(I) representation gave a MAE of 0.86 kcal/mol (model 5), which was 26% smaller than that of the AA-AC baseline.

Further exploring the use of the AABBA(I) kernel, we investigated how accuracy could be improved by fine-tuning other parameters. First, we considered other property operators and, in particular, the use of deltametric (property differences) and ratiometric (property ratios) ACs for the electronegativity and covalent radius, respectively (models 6 and 7 in Table 1). These operators encoded local variations in bond polarization and relative atomic size but, in practice, neither of them yielded lower MAEs. Next, we replaced the generic properties with the NBO, which, with the full origin, yielded the second lowest MAE in the series of numerical experiments: 0.76 kcal/mol (model 9). With the metal-centered origin, which is more useful from the perspective of explainability, the MAE could be reduced to 0.84 kcal/mol after increasing the maximum depth of the representation to six (model 12). In a final experiment, we enriched the most accurate representation by adding whole-graph properties but the increase in accuracy was very small (model 13).

**Figure 4:**
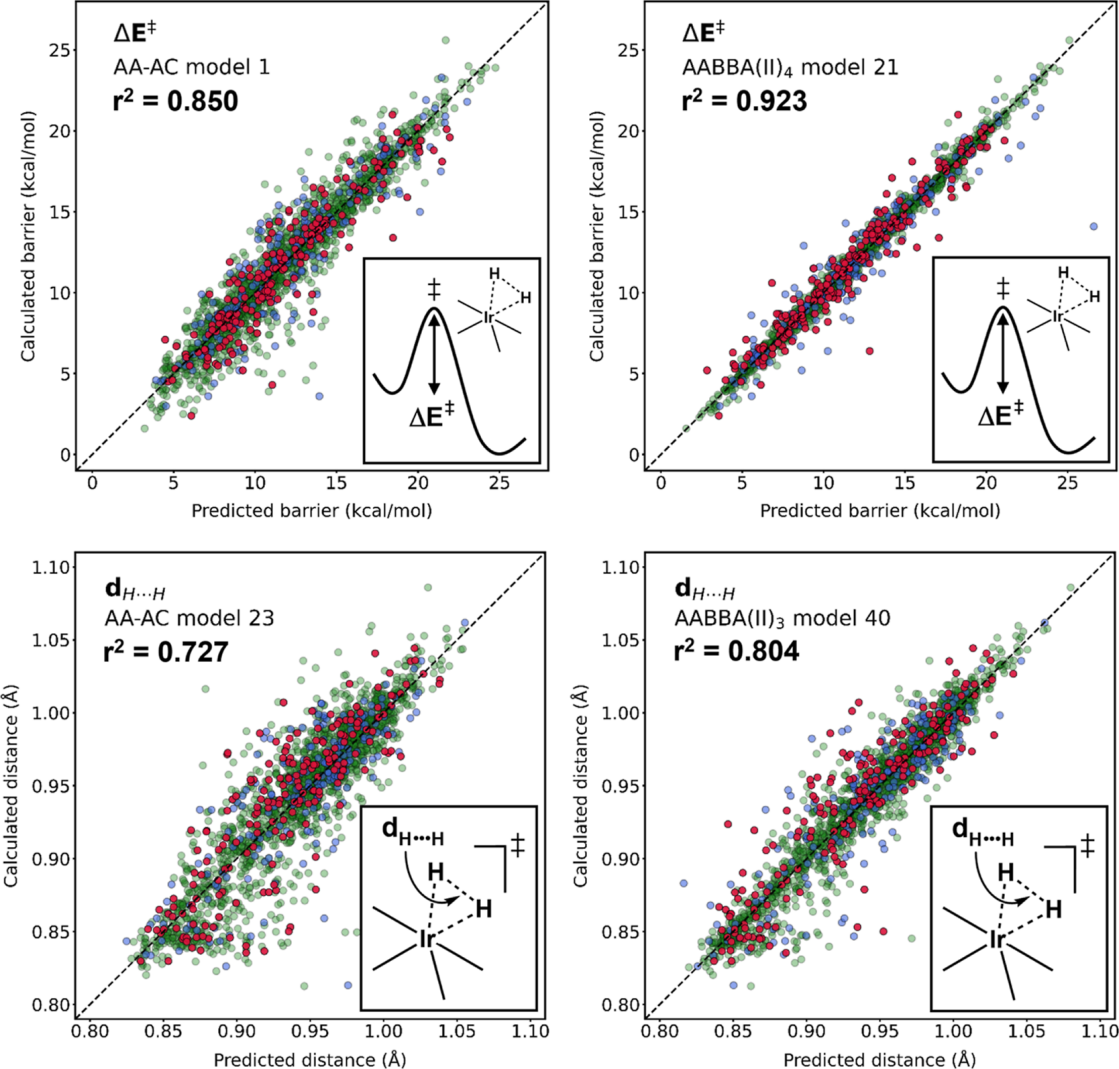
Pair plots showing the correlation between the DFT-calculated and NN-predicted energy barrier (Δ*E*^‡^) and breaking H···H bond distance (*d*_H···H_) of the Vaska’s data set. All models refer to Tables 1 and 2. Data points color code: green (training), blue (validation), and red (test).

The AABBA(II) kernel also outperformed the AA-AC baseline, reducing the MAE to an extent similar to AABBA(I), from 1.16 to 0.89 kcal/mol, using the AABBA(II)_1_ kernel (model 14 in Table 1). In contrast with AABBA(I), the use of NBO properties gave the highest accuracy when the AABBA(II)_4_ kernel was combined with the metal-centered origin and a maximum depth of five, yielding a MAE of 0.81 kcal/mol (model 21). Though it did not achieve the lowest MAE of the series, a significant advantage of this representation is its low dimensionality (129) relative to its AABBA(I) equivalent (298; model 11). The pair plots in Figure 4 show the performance of this AABBA(II) model relative to the AA-AC baseline.

### Transition State Distance

The prediction of the H···H bond distance was explored following the same systematic approach (Table 2). Using also the metal-centered autocorrelation of the generic properties at a maximum depth of three, the graph kernels including only atom-atom or bond-bond terms, i.e. AA-, -, and BB-AC, yielded similar accuracies, with MAE differences smaller than 0.002 Å (models 23–25). In contrast with the prediction of the energy barriers (Table 1), where AA-AC was the most accurate of these three kernels, the lowest MAE was hereby achieved with -AC (2.09 × 10^–2^ Å; model 25). This observation aligns with the notion that bond properties are crucial in predicting the distance, thereby contributing to improved results. The BA-AC kernel was also tested but it exhibited poorer performance (model 26).

Keeping the same properties, operator, origin, and depth, and expanding the autocorrelations with the AABBA(I) kernel, the MAE was reduced to 1.93 × 10^–2^ Å (model 27 in Table 2). Interestingly, by replacing the product autocorrelation of the electronegativity with its deltametric counterpart, the MAE was further minimized down to 1.87 × 10^–2^ Å (model 30), which was the lowest achieved with this kernel, showing the value of encoding bond polarity with electronegativity differences. Next, we moved to NBO properties, which, unlike the prediction of the energy barriers (Table 1), did not improve the results. The model closest in accuracy (35) used full autocorrelations with a maximum depth of five, thus producing large input vectors (dim = 316).

The lower-dimensionality vectors given by the AABBA(II) kernel also showed better performance with the generic properties than with the NBO (Table 2). The most accurate model in this series yielded MAE = 1.72 × 10^–2^ Å (model 40) using the AABBA(II)_3_ representation, which uses covalent radius instead of optimized interatomic distances, being thus geometry-agnostic. AABBA(II)_3_ reduced the MAE of the AA-AC baseline by 18% (see Figure 4 for further comparison). Increasing the maximum depth to six (models 44–46) or adding whole-graph properties (model 47) did not improve accuracy any further, suggesting that the H···H distance is dominated by local rather than global effects. Further, the increase of the depth could also yield noise, introducing detrimental effects on accuracy.

## Dimensionality Reduction by Feature Relevance

The results in Tables 1 and 2 showed that the performance of the NN models is sensitive to the nature of the graph kernel, as well as parameters like the origin and depth of the representations. The manual adjustment of these variables is challenging and, beyond domain knowledge and heuristics like, for example, using larger depths to capture remoter effects, optimal solutions could be easily missed. From this perspective, the comparison of the results obtained with the AABBA(I) and AABBA(II) kernels suggested that dimensionality reduction could be an appropriate strategy.

### Gradient Boosting Machines

We explored feature selection based on ensemble models by defining autocorrelation vectors with maximal dimensionality (MD), which were used to train GBM models predicting the Vaska’s data set energy barriers and breaking H···H bond distances. The GBM results were analyzed with the double aim of (1) comparing the different autocorrelation features from the perspective of their importance in the predictions, and (2) making a selection of these features for dimensionality-reduced ML models, which are presented in the next two sections.

The length of the MD autocorrelation vectors was maximized with the AABBA(I) graph kernel (Figure 2), using both the full and metal-centered origins, with a maximum depth of six, and all four arithmetic operators. All these terms were concatenated to form the final *v*_AABBA_^I,MD^ representation, which was either an 671- or 2750-dimensional vector, depending on whether the generic or NBO properties, respectively, were used to compute the autocorrelations. These dimensionalities are between six- and nine-times larger than those of the vectors fed to the NN models in Tables 1 and 2.

Regression trees (RTs) were used as base learners in the GBM ensemble models, which included 1000 RTs with a maximum depth of 5. The models were trained by minimizing the MSE loss with a learning rate of 0.05, and were tested by 5-fold cross-validation. Table 3 shows the performance of the GBMs in the prediction of the Vaska’s energy barriers and H···H distances. For both regression tasks, the NBO-informed representations gave higher accuracies than those based on generic properties. Another interesting observation is that the full *v*_AABBA_^I,MD^ vector achieved higher accuracies than the , BA, and AA alone, though the latter performed at nearly the same level when using NBO properties. The most accurate models yielded errors that were remarkably low for the barrier (MAE = 0.77 kcal/mol, *r*^2^ = 0.940) and moderate for the distance (MAE = 1.67 × 10^–2^ Å, *r*^2^ = 0.784). The latter model was the most accurate of the series reported in this work for the prediction of distances with NBO properties. Adding further context to the magnitude of these errors, the values of the two targets predicted were within the ranges [1.6–25.6] kcal/mol (barriers) and [0.81–1.09] Å (distances). Since the dimensions of AABBA autocorrelation vectors could be redundant to a significant extent, we explored how the selection of the most relevant features by the GBMs can be leveraged in dimensionality reduction.

**Table 3:** Average and Lowest Test Errors in the Prediction of the Vaska’s Dataset Energy Barrier (Δ*E*^‡^) and H···H Distance (*d*_H···H_) with GBM Models^a^

target	input			average error^b^		lowest error^b^	
	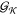	prop	dim^c^	MAE	*r*^2^	MAE	*r*^2^
**Δ*E***^**‡**^	**I**	**P**^d^	**671**	**1.00 ± 0.05**	**0.891** ± **0.004**	**0.91**	**0.919**
Δ*E*^‡^	AA	P^d^	223	1.09 ± 0.05	0.869 ± 0.02	1.01	0.896
Δ*E*^‡^	BB	P^d^	188	1.20 ± 0.04	0.858 ± 0.016	1.14	0.860
Δ*E*^‡^	BA	P^d^	260	1.29 ± 0.02	0.840 ± 0.018	1.24	0.867
***d***_**H···H**_	**I**	**P**^d^	**671**	**1.86 × 10^–2^ ± 7 × 10^–4^**	**0.738** ± **0.022**	**1.73 × 10^–2^**	**0.760**
*d*_H···H_	AA	P^d^	223	2.00 × 10^–2^ ± 5 × 10^–4^	0.711 ± 0.021	1.89 × 10^–2^	0.750
*d*_H···H_	BB	P^d^	188	2.06× 10^–2^ ± 6 × 10^–4^	0.706 ± 0.021	1.97 × 10^–2^	0.732
*d*_H···H_	AB	P^d^	260	2.16 × 10^–2^ ± 7 × 10^–4^	0.671 ± 0.025	2.05 × 10^–2^	0.703
**Δ*E***^**‡**^	**I**	**NBO**^e^	**2750**	**0.84 ± 0.04**	**0.920** ± **0.016**	**0.77**	**0.940**
Δ*E*^‡^	AA	NBO^e^	680	0.85 ± 0.04	0.918 ± 0.019	0.79	0.942
Δ*E*^‡^	BB	NBO^e^	1068	1.05 ± 0.02	0.888 ± 0.012	1.02	0.902
Δ*E*^‡^	BA	NBO^e^	1002	1.07 ± 0.04	0.877 ± 0.018	0.98	0.902
***d***_**H···H**_	**I**	**NBO**^e^	**2750**	**1.79 × 10^–2^ ± 6 × 10^–4^**	**0.753** ± **0.018**	**1.67 × 10^–2^**	**0.784**
*d*_H···H_	AA	NBO^e^	680	1.80 × 10^–2^ ± 6 × 10^–4^	0.748 ± 0.022	1.71 × 10^–2^	0.777
*d*_H···H_	BB	NBO^e^	1068	1.99 × 10^–2^ ± 9 × 10^–4^	0.711 ± 0.032	1.86 × 10^–2^	0.762
*d*_H···H_	BA	NBO^e^	1002	1.95 × 10^–2^ ± 6 × 10^–4^	0.708 ± 0.025	1.85 × 10^–2^	0.739

a The inputs passed to the models were *v*_AABBA_^I,MD^ vectors defined with different property types (prop) yielding different (maximal) dimensionalities (dim). The mean absolute errors (MAEs) are given in kcal/mol for the barriers and Å for the distances.

b From 5-fold cross-validation.

c After removing redundant dimensions.

d I.e. P_A_, P_B_, and P_AB_ generic property sets.

e I.e. P_A,NBO_, P_B,NBO_, and P_AB,NBO_ NBO property sets. See the Supporting Information for further details.

**Figure 5:**
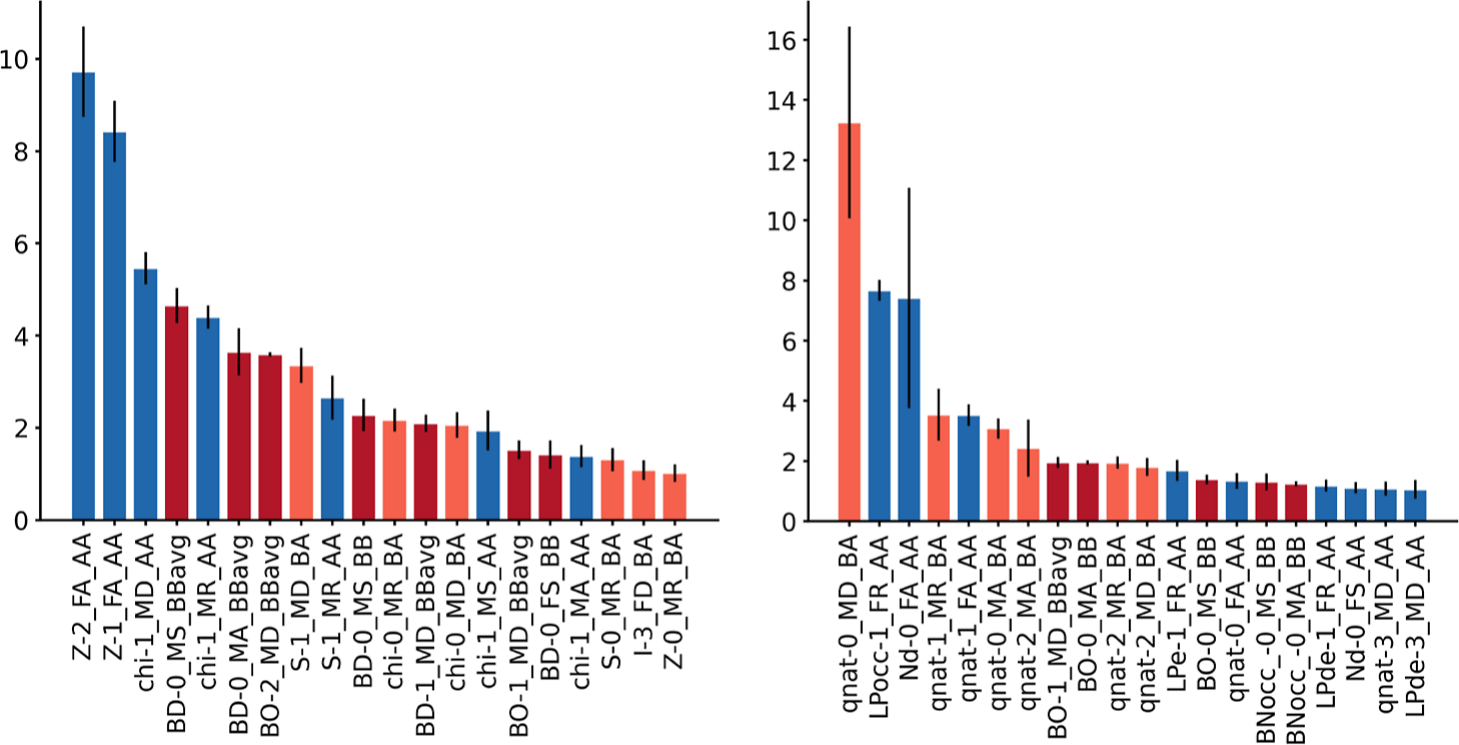
Relevances (*y*-axes, in %) of the 20 most important AABBA components (*x*-axes) from the GBM models predicting the Vaska’s energy barriers. Bar color code: blue (AA-AC), purple (BB-AC and -AC), and orange (BA-AC).

**Figure 6:**
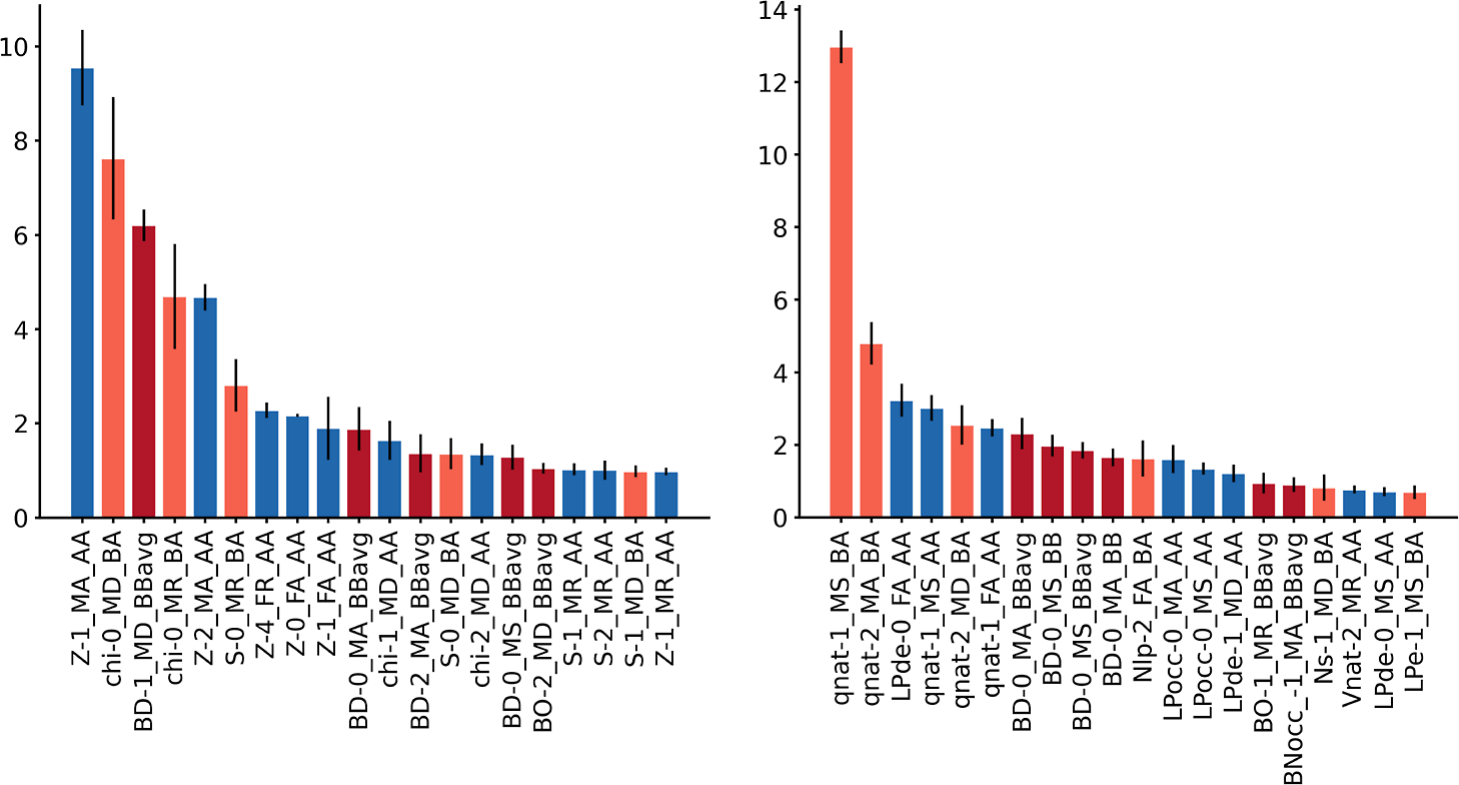
Relevances (*y*-axes, in %) of the 20 most important AABBA components (*x*-axes) from the GBM models predicting the Vaska’s breaking H···H distances. Bar color code: blue (AA-AC), purple (BB-AC and -AC), and orange (BA-AC).

The relevance of the features, i.e. the many dimensions of the *v*_AABBA_^I,MD^ autocorrelation vectors, was computed using the Friedman MSE criterion.^66^ This criterion exploits the ensemble nature of the GBMs, quantifying the reduction of the residual sum of squares of the model by any given feature relative to the total. Figures 5 and 6 show the 20 most relevant features in the GBM models predicting the barriers and distances, respectively, from the generic and NBO representations. In all cases, the three atom–atom, bond–bond, and bond–atom components of the AABBA(I) graph kernel yielded features of high relevance. For example, in the prediction of the energy barrier with generic properties, there is a similar number of AA, BB, and BA features (i.e., 7, 7, and 6, respectively) among the 20 most relevant, which is also true in the other three cases. Table 4, which collects and describes only the 5 most relevant features, also shows some interesting trends. For the barriers, there is a mix of full and metal-centered features, which, with the generic properties, refer to chemical composition (*Z*) and electronegativity (χ), whereas, with the NBO properties, refer to natural electron counts and charges (*q*_Nat_). There is also a mix of full and metal-centered features, which is consistent with the influence of both local and global effects on the oxidative addition barrier. Further, features like the bond–atom metal-centered charges can be directly related to the critical role played by the electron density of the metal center. For the distances, most features are metal-centered at depth zero or one, in line with the local nature of the H···H bond cleavage, which takes place within the first coordination sphere of the metal center. As for the barrier, the generic features involve mostly *Z* and χ, whereas the NBO are dominated by *q*_Nat_. Regardless of the target, the product autocorrelation operator is the most common, appearing in half of the features, whereas the other half is diverse, including the ratiometric, symmetric, and deltametric operators.

**Table 4:** Five Most Relevant Features in the Prediction of the Vaska’s Dataset Energy Barrier (Δ*E*^‡^) and Breaking H···H Distance (*d*_H···H_) with Generic (P) and Natural Bond Orbital (NBO) Properties^a^

rank	feature label	feature description
Target = Δ*E*^‡^, Properties = P		
1	Z-2_FA_AA	full atom–atom autocorrelation of atomic number at depth 2
2	*Z*-1_FA_AA	full atom–atom autocorrelation of atomic number at depth 1
3	χ-1_MD_AA	metal-centered atom-atom deltametric of electronegativity at depth 1
4	BD-0_MS_BB̅	metal-centered averaged bond-bond summetric of distance at depth 0
5	χ-1_MR_AA	metal-centered atom-atom ratiometric of electronegativity at depth 1
Target = Δ*E*^‡^, Properties = NBO		
1	*q*_Nat_-0_MD_BA	metal-centered bond–atom deltametric of natural charge at depth 0
2	LP_Occ_-1_FR_AA	full atom–atom ratiometric of lone-pair occupancies at depth 1
3	*N*_d_-0_FA_AA	full atom–atom autocorrelation of d-electron count at depth 0
4	*q*_Nat_-1_MR_BA	metal-centered bond–atom ratiometric of natural charge at depth 1
5	*q*_Nat_-1_FA_AA	full atom–atom autocorrelation of natural charge at depth 1
Target = *d*_H··· H_, Properties = P		
1	*Z*-1_MA_AA	metal-centered atom–atom autocorrelation of atomic number at depth 1
2	χ-0_MD_BA	metal-centered bond–atom deltametric of electronegativity at depth 0
3	BD-1_MD_BB̅	metal-centered averaged bond–bond deltametric of distance at depth 0
4	χ-0_MR_BA	metal-centered bond–atom ratiometric of electronegativity at depth 0
5	*Z*-2_MA_AA	metal-centered atom–atom autocorrelation of atomic number at depth 2
Target = d_H··· H_, Properties = NBO		
1	*q*_Nat_-1_MS_BA	metal-centered bond–atom summetric of natural charge at depth 1
2	*q*_Nat_-2_MA_BA	metal-centered bond–atom autocorrelation of natural charge at depth 2
3	LP_ΔE_-0_FA_AA	full atom–atom autocorrelation of lone-pair energy gap at depth 0
4	*q*_Nat_-1_MS_AA	metal-centered atom–atom summetric of natural charge at depth 1
5	*q*_Nat_-2_MD_BA	metal-centered bond–atom deltametric of natural charge at depth 2

a The Rank Refers to Feature Relevance as Derived From the GBM Models.

### Gaussian Processes

The use of AABBA representations with reduced dimensionality was investigated with GPs predicting the Vaska’s energy barriers and breaking H···H distances. These models were based on a composed Linear-RBF kernel (; RBF = radial basis function) in which both covariance functions were multiplied; i.e.
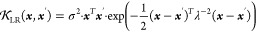
1where (*x*, *x*′) is a pair of data points, σ^2^ is the variance, and λ is the length-scale of the kernel. Preliminary studies showed that these two kernels perform at a lower level when used separately in these regression tasks.

**Figure 7:**
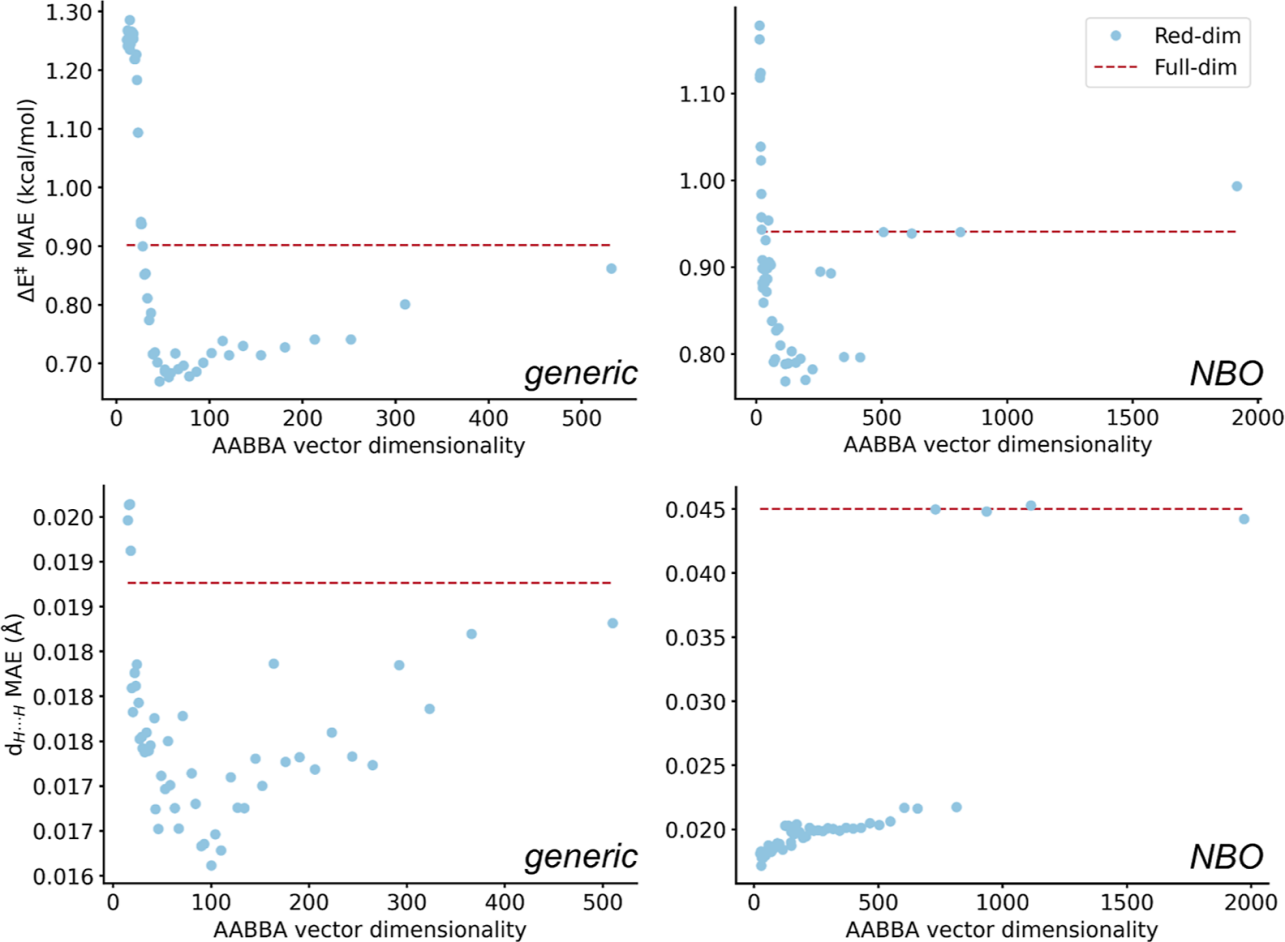
Influence of reducing the dimensionality of the AABBA representation in the MAEs of the GP predicting the Vaska’s barriers (Δ*E*^‡^; top) and distances (*d*_H···H_; bottom) from generic (left) and NBO (right) properties. The legend applies to all four plots.

The *v*_AABBA_^I,MD^ vectors with maximal dimensionality were simplified by gradually removing terms according to the accumulated relevance found with the GBM models (Tables S5 and S6). MAEs were computed for each pruned representation, using that of the full *v*_AABBA_^I,MD^ vector as baseline. In the prediction of Δ*E*^‡^ with generic properties (Figure 7), we observed a gentle decrease of the MAE until a reduced dimensionality of ∼50, followed by a steep increase of the MAE at smaller dimensionalities. The top-performance model used a 46-dimensional AABBA representation (80% accumulated relevance), in which 59% of the terms were either BB or BA (Table 5)—Remarkably, with MAE = 0.67 kcal/mol and *r*^2^ = 0.947, this GP model was the most accurate of the series reported in this work. With the NBO features, the convergence of the MAE with the reduction of the dimensionality was less stable and two minima were observed, as clearly shown by the accumulated relevance plots (Figure S8). The lowest MAE model was based on a 115-dimensional AABBA vector (86% accumulated relevance) in which the BB and BA terms amounted 54% of the total dimensions. With MAE = 0.77 kcal/mol and *r*^2^ = 0.930, this model performed at a level similar to that of the most accurate NNs found in the systematic study (e.g., model 9 in Table 1).

**Table 5:**
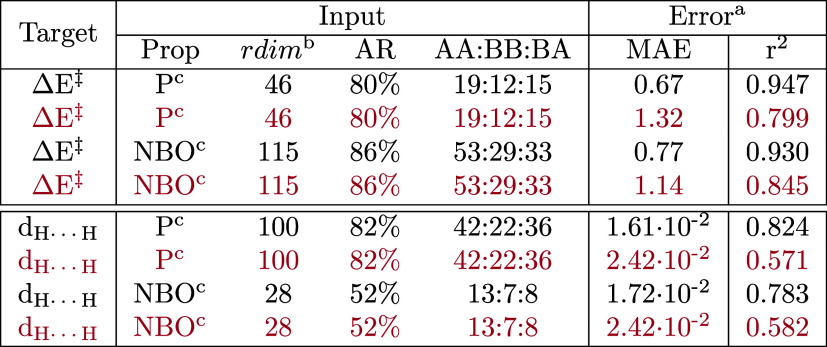
Test Errors in the Prediction of the Vaska’s Dataset Energy Barrier (Δ*E*^‡^) and H···H Distance (*d*_H···H_) with Gaussian Processes^a^

a The inputs passed to the models were *v*_AABBA_^I,MD^ vectors defined with different property types (prop) after being pruned to a reduced dimensionality (rdim) based on the accumulated relevance (AR) found with the GBM models. The mean absolute errors (MAEs) are given in Kcal/mol for the barriers and Å for the distances.

b From ten repetitions with a training:validation:test split of 80:10:10 (black) or 20:40:40 (purple).

c After pruning the AABBA representation with respect to the GBM relevances.

d I.e. P_A_, P_B_, and P_AB_ generic property sets.

e I.e. P_A,NBO_, P_B,NBO_, and P_AB,NBO_ NBO property sets. See the Supporting Information for further details.

In the prediction of the distances (Figure 7), the generic properties showed a trend that was less stable and yet similar to that observed for the barriers. The MAE was minimized to 1.61 × 10^–2^ Å (*r*^2^ = 0.824) with a simplified input of 100 dimensions (82% accumulated relevance), of which 58% were either BB or BA (Table 5). This model was also the most accurate of the series, thus showing that the use of Gaussian processes with reduced AABBA representations computed from generic properties is a powerful approach to the prediction of the Vaska’s barriers and distances. With the NBO properties, there was a sharp decrease in the MAE at ∼700 dimensions, reaching a minimum at 1.72 × 10^–2^ Å (*r*^2^ = 0.783) with a 28-dimensional vector, in which the BB and BA terms were 54% of the representation. This distinct behavior might be caused by the use of a representation that is very rich in electronic structure features to predict a single target that is purely geometric.

The data efficiency of the GP models was also investigated with a training/validation/test data split of 20:40:40 (Table 5). Whereas the accuracy of the resulting models was reasonable for the prediction of the barriers, with MAE = 1.1–1.4 kcal/mol and *r*^2^ = 0.79–0.85, the performance in the prediction of the distances was rather poor, with MAE = 2.42 × 10^−2^ Å and *r*^2^ < 0.6, suggesting that the latter regression task must involve further changes in the models before dimensionality reduction can be leveraged efficiently in a small-data training context.

### Neural Networks

Dimensionality reduction was also explored in the prediction of the Vaska’s barriers and distances with NN models. Due to higher computational cost, the recalculation of the GP scatter plots of Figure 7 for the NNs was performed with a smaller resolution (Figure S9). Since the trends observed with both models were similar, the NNs were fed with *v*_AABBA_^I,MD^ vectors pruned down to the same optimally reduced dimensions found with the GP (Tables 5 and 6). For the sake of comparability, we used the same NN hyperparameters that yielded the results shown in Tables 1 and 2.

**Table 6:**
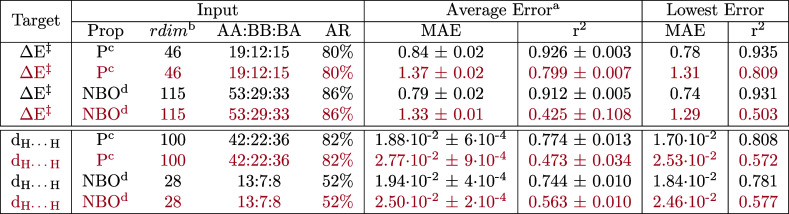
Test Errors in the Prediction of the Vaska’s Dataset Energy Barrier (Δ*E*^‡^) and H···H Distance (*d*_H···H_) with NNs^a^

a The Inputs passed to the models were *v*_AABBA_^*I*,*MD*^ vectors defined with different property types (prop) after being pruned to a reduced dimensionality (rdim) based on the accumulated relevance (AR) and MAEs found with the GBM and GP models, respectively. The mean absolute errors (MAEs) are given in Kcal/mol for the barriers and Å for the distances.

b From ten repetitions with a training/validation/test split of 80:10:10 (black) or 20:40:40 (purple).

c After pruning the AABBA representation with respect to the GBM relevances.

d I.e. P_A_, P_B_, and P_AB_ generic property sets.

e I.e. P_A,NBO_, P_B,NBO_, and P_AB,NBO_ NBO property sets. See the Supporting Information for further details.

In the prediction of the barriers and distances with generic properties, the lowest errors, i.e. MAE = 0.78 kcal/mol and 1.70 × 10^–2^ Å (Table 6), respectively, were either larger than or similar to those found with the GP (Table 5) and systematic NN (Table 1) models. In the predictions based on NBO properties, the model yielding MAE = 0.74 kcal/mol and *r*^2^ = 0.931 for the prediction of the barriers was one of the most accurate of the series reported in this work. For the distances, the performance level was lower than that of the GP and higher than that of the systematic NNs, with MAE = 1.84 × 10^–2^ (Table 2). In a final experiment, we explored small-data training with the reduced representations using a training/validate/test ratio of 20:40:40, and, in line with the GP results, moderate accuracies were only achieved in the prediction of the energy barriers.

In general, dimensionality could be significantly reduced to increase accuracy with both the GP and NN models, in line with previous observations made for similar ML models leveraging only atom-atom autocorrelations.^52^ To some extent, this behavior could be expected in a framework in which the dimensionality of the largest representations is similar to the total number of data points available for training the models. Compared to the systematic models shown in Tables 1 and 2, in which the inputs were manually set, the GBM-engineered AABBA representations achieved higher accuracies in the prediction of both targets with both property types, though often by a small margin. This simplification of the *v*_AABBA_^I,MD^ vector did not alter the nature of the properties yielding the lowest MAEs; i.e. generic for the prediction of the distances and NBO for the barriers (Tables 5 and 6). Another common trend between the GP and NNs is that the most accurate models using generic properties needed more dimensions for predicting the distances than the barriers, whereas the opposite was true with the NBO properties. These results also showed that, for these regression tasks, accumulating all possible AABBA terms into a large-dimensional vector is not an efficient strategy. Interestingly, in all cases, the bond-bond and bond-atom terms extending the conventional atom-atom autocorrelations accounted for 50–60% of the reduced representation.

## Conclusions

This work showed how to extract vectors of features from molecular graphs attributed with both atomic and bond properties, following these two distinct approaches: (1) concatenation of atom–atom, bond–bond, and bond–atom autocorrelation terms, as implemented in the AABBA(I) kernel; and (2) merging atom and bond properties into vectors that were subsequently autocorrelated into a smaller dimensionality representation, as implemented in the AABBA(II) kernel. The AABBA(I) kernel was implemented in a modular way, allowing for using the AA-AC, BB-AC, -AC, and BA-AC autocorrelations as either stand-alone representations or in any of their possible combinations.

The vectors generated by the AABBA kernels from the molecular graphs were used as input for the prediction of the energy barriers and breaking H···H distances of the Vaska’s data set. In a systematic approach gradually adding complexity and dimensionality to the input of NN models, we found that, when used independently, the bond-bond kernels, especially the -AC, were performing at a level similar to that of the traditional atom-atom kernels. Once all these kernels were combined into AABBA(I), the resulting representation outperformed the AA-AC by a significant margin. High accuracies could be obtained with this kernel using both generic and NBO properties. In general, the influence of the origin, property operator, and maximal depth on the performance of the NNs was either moderate or small. Interestingly, the accuracies achieved with the lower-dimension AABBA(II) kernel were similar (barrier) or higher (distance) than those of AABBA(I), showing the potential benefits of dimensionality reduction in this context.

After maximizing the dimensionality of the representation with the AABBA(I) kernel, GBM models were used to quantify feature relevance in the prediction of the Vaska’s barriers and distances. Among the 20 most important features, ca. half of them were extracted by the BB-AC, -AC, and BA-AC components of the kernel, showing the advantage of leveraging bond properties in these regression tasks. Feature relevance also allowed for interpreting the model predictions, showing that, whereas the barriers were mostly correlated to global features encoding electronic structure information, distances were more dependent on local metal-centered features encoding bond information. Dimensionality reduction was also explored with GP and NN models in which the dimensions of the AABBA(I) representation were gradually removed according to the feature relevances found by the GBMs. This approach proved efficient, yielding many of the most accurate models reported in this study. In line with the GBM results, ∼ 50% of the dimensions of the reduced autocorrelation vectors were extracted by a kernel operating on bond properties.

In summary, this work showed that the Moreau-Broto AA-AC autocorrelation kernel for molecular graphs can be extended to include bond-bond and bond-atom terms, increasing the accuracy of the ML models in which the resulting vectors are leveraged as input. For optimal results with the AABBA(I) kernel, we recommend performing dimensionality reduction to achieve higher accuracies. If this feature engineering approach is too involved for the application intended, the AABBA(II) kernel does virtually the same in an implicit and simple manner, achieving similar accuracies. Further, if accuracy is not critical, the stand-alone use of the bond-bond kernels, in particular the -AC, can also yield satisfactory results at a level close to that of the conventional AA-AC kernel and with the advantage of using a smaller dimensionality representation.

In perspective, the AABBA graph kernel can be useful in the exploration of the chemical space by means of active learning methods, covering also the transition metal complexes. Further, the modular nature of AABBA could be easily adapted to the featurization of materials built with repeating molecular blocks; for example: nanoporous materials like MOFs. We also showed that the use of NBO properties can boost the accuracy of machine learning models by providing additional information based on quantum physics, which can also be leveraged for other purposes including, for example, the encoding of conformational effects or the interpretation of model predictions.^65^ However, it should be noted that this data has a significant computational cost, though much lower than that of first and second derivatives of the energy in, for example, transition state optimizations. In this regard, it can be worth exploring the use of NBO properties obtained at the lowest possible levels of theory; for example: DFT with the local spin density approximation or semiempirical methods.^67^ Another valuable extension of the AABBA representation can be the inclusion of the metal oxidation state by adding it as an additional atomic property or by appending a categorical encoding of it to the autocorrelation vectors.

## Data Availability

The open repository https://github.com/uiocompcat/AABBA provides access to the code of the AABBA graph kernels and ML models, as well as to the u-NatQG graphs of the Vaska’s complex data set and the associated AABBA vectors. The HyDGL code used to generate the u-NatQG graphs is available from https://github.com/uiocompcat/HyDGL.

## Supporting Information Available

Detailed description of the AABBA graph kernels, including the underlying equations, dimensionality of the resulting vectors, definition of the metal-centered edge origin, systematic lists of generic and NBO properties, metal-centered depth distribution, computational details of the NN, GBM, and GP models, and further information on feature relevance and dimensionality reduction (PDF)

## References

[ref1] Meyer B.; Sawatlon B.; Heinen S.; von Lilienfeld O. A.; Corminboeuf C. Machine learning meets volcano plots: computational discovery of cross-coupling catalysts. Chem. Sci. 2018, 9, 7069–7077. 10.1039/C8SC01949E. 30310627 PMC6137445

[ref2] Wellendorff J.; Lundgaard K. T.; Mogelhoj A.; Petzold V.; Landis D. D.; Norskov J. K.; Bligaard T.; Jacobsen K. W. Density functionals for surface science: Exchange-correlation model development with Bayesian error estimation. Phys. Rev. B 2012, 85, 235149 10.1103/PhysRevB.85.235149.

[ref3] Tran K.; Ulissi Z. W. Active learning across intermetallics to guide discovery of electrocatalysts for CO2 reduction and H2 evolution. Nat. Catal. 2018, 1, 696–703. 10.1038/s41929-018-0142-1.

[ref4] Karl T. M.; Bouayad-Gervais S.; Hueffel J. A.; Sperger T.; Wellig S.; Kaldas S. J.; Dabranskaya U.; Ward J. S.; Rissanen K.; Tizzard G. J.; et al. Machine Learning-Guided Development of Trialkylphosphine Ni-(I) Dimers and Applications in Site-Selective Catalysis. J. Am. Chem. Soc. 2023, 145, 15414–15424. 10.1021/jacs.3c03403. 37411044

[ref5] Nandy A.; Duan C.; Goffinet C.; Kulik H. J. New Strategies for Direct Methane-to-Methanol Conversion from Active Learning Exploration of 16 Million Catalysts. JACS Au 2022, 2, 1200–1213. 10.1021/jacsau.2c00176. 35647589 PMC9135396

[ref6] Gensch T.; dos Passos Gomes G.; Friederich P.; Peters E.; Gaudin T.; Pollice R.; Jorner K.; Nigam A.; Lindner-D’Addario M.; Sigman M. S.; Aspuru-Guzik A. A Comprehensive Discovery Platform for Organophosphorus Ligands for Catalysis. J. Am. Chem. Soc. 2022, 144, 1205–1217. 10.1021/jacs.1c09718. 35020383

[ref7] Pollice R.; Dos Passos Gomes G.; Aldeghi M.; Hickman R. J.; Krenn M.; Lavigne C.; Lindner-D’Addario M.; Nigam A.; Ser C. T.; Yao Z.; Aspuru-Guzik A. Data-Driven Strategies for Accelerated Materials Design. Acc. Chem. Res. 2021, 54, 849–860. 10.1021/acs.accounts.0c00785. 33528245 PMC7893702

[ref8] Butler K. T.; Davies D. W.; Cartwright H.; Isayev O.; Walsh A. Machine learning for molecular and materials science. Nature 2018, 559, 547–555. 10.1038/s41586-018-0337-2. 30046072

[ref9] Schmidt J.; Marques M. R. G.; Botti S.; Marques M. A. L. Recent advances and applications of machine learning in solid-state materials science. Npj Comput. Mater. 2019, 5, 83 10.1038/s41524-019-0221-0.

[ref10] Severson K. A.; Attia P. M.; Jin N.; Perkins N.; Jiang B.; Yang Z.; Chen M. H.; Aykol M.; Herring P. K.; Fraggedakis D.; et al. Data-driven prediction of battery cycle life before capacity degradation. Nat. Energy 2019, 4, 383–391. 10.1038/s41560-019-0356-8.

[ref11] Ward L.; Agrawal A.; Choudhary A.; Wolverton C. A general-purpose machine learning framework for predicting properties of inorganic materials. npj Comput. Mater. 2016, 2, 16028 10.1038/npjcompumats.2016.28.

[ref12] Tshitoyan V.; Dagdelen J.; Weston L.; Dunn A.; Rong Z. Q.; Kononova O.; Persson K. A.; Ceder G.; Jain A. Unsupervised word embeddings capture latent knowledge from materials science literature. Nature 2019, 571, 95–98. 10.1038/s41586-019-1335-8. 31270483

[ref13] Jumper J.; Evans R.; Pritzel A.; Green T.; Figurnov M.; Ronneberger O.; Tunyasuvunakool K.; Bates R.; Žídek A.; Potapenko A.; et al. Highly accurate protein structure prediction with AlphaFold. Nature 2021, 596, 583–589. 10.1038/s41586-021-03819-2. 34265844 PMC8371605

[ref14] Vamathevan J.; Clark D.; Czodrowski P.; Dunham I.; Ferran E.; Lee G.; Li B.; Madabhushi A.; Shah P.; Spitzer M.; et al. Applications of machine learning in drug discovery and development. Nat. Rev. Drug Discovery 2019, 18, 463–477. 10.1038/s41573-019-0024-5. 30976107 PMC6552674

[ref15] Lo Y. C.; Rensi S. E.; Torng W.; Altman R. B. Machine learning in chemoinformatics and drug discovery. Drug Discovery Today 2018, 23, 1538–1546. 10.1016/j.drudis.2018.05.010. 29750902 PMC6078794

[ref16] Altae-Tran H.; Ramsundar B.; Pappu A. S.; Pande V. Low Data Drug Discovery with One-Shot Learning. ACS Cent. Sci. 2017, 3, 283–293. 10.1021/acscentsci.6b00367. 28470045 PMC5408335

[ref17] Yang X.; Wang Y. F.; Byrne R.; Schneider G.; Yang S. Y. Concepts of Artificial Intelligence for Computer-Assisted Drug Discovery. Chem. Rev. 2019, 119, 10520–10594. 10.1021/acs.chemrev.8b00728. 31294972

[ref18] Lavecchia A. Machine-learning approaches in drug discovery: methods and applications. Drug Discovery Today 2015, 20, 318–331. 10.1016/j.drudis.2014.10.012. 25448759

[ref19] Mater A. C.; Coote M. L. Deep Learning in Chemistry. J. Chem. Inf. Model. 2019, 59, 2545–2559. 10.1021/acs.jcim.9b00266. 31194543

[ref20] Goh G. B.; Hodas N. O.; Vishnu A. Deep learning for computational chemistry. J. Comput. Chem. 2017, 38, 1291–1307. 10.1002/jcc.24764. 28272810

[ref21] Popova M.; Isayev O.; Tropsha A. Deep reinforcement learning for de novo drug design. Sci. Adv. 2018, 4, eaap7885 10.1126/sciadv.aap7885. 30050984 PMC6059760

[ref22] Simm G. N.; Reiher M. Error-Controlled Exploration of Chemical Reaction Networks with Gaussian Processes. J. Chem. Theory Comput. 2018, 14, 5238–5248. 10.1021/acs.jctc.8b00504. 30179500

[ref23] Deringer V. L.; Bartók A. P.; Bernstein N.; Wilkins D. M.; Ceriotti M.; Csányi G. Gaussian Process Regression for Materials and Molecules. Chem. Rev. 2021, 121, 10073–10141. 10.1021/acs.chemrev.1c00022. 34398616 PMC8391963

[ref24] Shields B. J.; Stevens J.; Li J.; Parasram M.; Damani F.; Alvarado J. I. M.; Janey J. M.; Adams R. P.; Doyle A. G. Bayesian reaction optimization as a tool for chemical synthesis. Nature 2021, 590, 89–96. 10.1038/s41586-021-03213-y. 33536653

[ref25] Zheng C.; Chen C.; Chen Y. M.; Ong S. P. Random Forest Models for Accurate Identification of Coordination Environments from X-Ray Absorption Near-Edge Structure. Patterns 2020, 1, 100013 10.1016/j.patter.2020.100013. 33205091 PMC7660409

[ref26] Sheridan R. P.; Wang w. M.; Liaw A.; Ma J.; Gifford E. M. Extreme Gradient Boosting as a Method for Quantitative Structure–Activity Relationships. J. Chem. Inf. Model. 2016, 56, 2353–2360. 10.1021/acs.jcim.6b00591. 27958738

[ref27] Li L.; Zhao Y.; Yu H.; Wang Z.; Zhao Y.; Jiang M. An XGBoost Algorithm Based on Molecular Structure and Molecular Specificity Parameters for Predicting Gas Adsorption. Langmuir 2023, 39, 6756–6766. 10.1021/acs.langmuir.3c00255. 37130050

[ref28] Chen S.; Nielson T.; Zalit E.; Skjelstad B. B.; Borough B.; Hirschi W. J.; Yu S.; Balcells D.; Ess D. H. Automated Construction and Optimization Combined with Machine Learning to Generate Pt(II) Methane C-H Activation Transition States. Top. Catal. 2022, 65, 312–324. 10.1007/s11244-021-01506-0.

[ref29] Mayr A.; Klambauer G.; Unterthiner T.; Hochreiter S. DeepTox: Toxicity Prediction using Deep Learning. Front. Environ. Sci. 2016, 3, 80 10.3389/fenvs.2015.00080.

[ref30] Irwin J. J.; Sterling T.; Mysinger M. M.; Bolstad E. S.; Coleman R. G. ZINC: A Free Tool to Discover Chemistry for Biology. J. Chem. Inf. Model. 2012, 52, 1757–1768. 10.1021/ci3001277. 22587354 PMC3402020

[ref31] Ramakrishnan R.; Dral P. O.; Rupp M.; von Lilienfeld O. A. Quantum chemistry structures and properties of 134 kilo molecules. Sci. Data 2014, 1, 140022 10.1038/sdata.2014.22. 25977779 PMC4322582

[ref32] Ruddigkeit L.; van Deursen R.; Blum L. C.; Reymond J.-L. Enumeration of 166 Billion Organic Small Molecules in the Chemical Universe Database GDB-17. J. Chem. Inf. Model. 2012, 52, 2864–2875. 10.1021/ci300415d. 23088335

[ref33] Collins C. R.; Gordon G. J.; von Lilienfeld O. A.; Yaron D. J. Constant size descriptors for accurate machine learning models of molecular properties. J. Chem. Phys. 2018, 148, 241718 10.1063/1.5020441. 29960361

[ref34] Huang B.; von Lilienfeld O. A. Communication: Understanding molecular representations in machine learning: The role of uniqueness and target similarity. J. Chem. Phys. 2016, 145, 161102 10.1063/1.4964627. 27802646

[ref35] De S.; Bartok A. P.; Csanyi G.; Ceriotti M. Comparing Molecules and Solids across Structural and Alchemical Space. Phys. Chem. Chem. Phys. 2016, 18, 13754–13769. 10.1039/C6CP00415F. 27101873

[ref36] Nandy A.; Duan C. R.; Taylor M. G.; Liu F.; Steeves A. H.; Kulik H. J. Computational Discovery of Transition-metal Complexes: From High-throughput Screening to Machine Learning. Chem. Rev. 2021, 121, 9927–10000. 10.1021/acs.chemrev.1c00347. 34260198

[ref37] Balcells D.; Skjelstad B. B. tmQM Dataset—Quantum Geometries and Properties of 86k Transition Metal Complexes. J. Chem. Inf. Model. 2020, 60, 6135–6146. 10.1021/acs.jcim.0c01041. 33166143 PMC7768608

[ref38] Duan C.; Nandy A.; Meyer R.; Arunachalam N.; Kulik H. J. A Transferable Recommender Approach for Selecting the Best Density Functional Approximations in Chemical Discovery. Nat. Comput. Sci. 2023, 3, 38–47. 10.1038/s43588-022-00384-0. 38177951

[ref39] Kneiding H.; Nova A.; Balcells D. Directional Multiobjective Optimization of Metal Complexes at the Billion-System Scale. Nat. Comput. Sci. 2024, 4, 263–273. 10.1038/s43588-024-00616-5. 38553635

[ref40] Weininger D. SMILES, a chemical language and information system. 1. Introduction to methodology and encoding rules. J. Chem. Inf. Comput. Sci. 1988, 28, 31–36. 10.1021/ci00057a005.

[ref41] Krenn M.; Häse F.; Nigam A.; Friederich P.; Aspuru-Guzik A. Self-referencing embedded strings (SELFIES): A 100% robust molecular string representation. Mach. Learn.: Sci. Technol. 2020, 1, 045024 10.1088/2632-2153/aba947.

[ref42] Lo A.; Pollice R.; Nigam A. K.; White A. D.; Krenn M.; Aspuru-Guzik A. Recent advances in the self-referencing embedded strings (SELFIES) library. Digital Discovery 2023, 2, 897–908. 10.1039/D3DD00044C. 38013816 PMC10408573

[ref43] Guan Y.; Coley C. W.; Wu H.; Ranasinghe D.; Heid E.; Struble T. J.; Pattanaik L.; Green W. H.; Jensen K. F. Regio-selectivity prediction with a machine-learned reaction representation and on-the-fly quantum mechanical descriptors. Chem. Sci. 2021, 12, 2198–2208. 10.1039/D0SC04823B. PMC8179287 34163985

[ref44] Boiko D. A.; Reschützegger T.; Sanchez-Lengeling B.; Blau S. M.; Gomes G. Advancing Molecular Machine (Learned) Representations with Stereoelectronics-Infused Molecular Graphs. arXiv 2024, arXiv:2408.04520 10.48550/arXiv.2408.04520.

[ref45] Kneiding H.; Balcells D. Deep Graph Learning for Molecules and Materials. Nordic Machine Intelligence 2023, 3, 8–14. 10.5617/nmi.10517.

[ref46] Gallegos L. C.; Luchini G.; St John P. C.; Kim S.; Paton R. S. Importance of Engineered and Learned Molecular Representations in Predicting Organic Reactivity, Selectivity, and Chemical Properties. Acc. Chem. Res. 2021, 54, 827–836. 10.1021/acs.accounts.0c00745. 33534534

[ref47] Kneiding H.; Lukin R.; Lang L.; Reine S.; Pedersen T. B.; de Bin R.; Balcells D. Deep Learning Metal Complex Properties with Natural Quantum Graphs. Digital Discovery 2023, 2, 618–633. 10.1039/D2DD00129B.

[ref48] Vargas S.; Gee W.; Alexandrova A. High-throughput quantum theory of atoms in molecules (QTAIM) for geometric deep learning of molecular and reaction properties. Digital Discovery 2024, 3, 987–998. 10.1039/D4DD00057A.

[ref49] Rupp M.; Schneider G. Graph Kernels for Molecular Similarity. Mol. Inf. 2010, 29, 266–273. 10.1002/minf.200900080. 27463053

[ref50] Todeschini R.; Consonni V. Handbook of Molecular Descriptors; Wiley VCH: Weinheim, Germany, 2008; Vol. 11. 10.1002/9783527613106.

[ref51] Moreau G.; Broto P. Autocorrelation of a topological structure: A new molecular descriptor. Nouv. J. Chim. 1980, 4, 359–360.

[ref52] Janet J. P.; Kulik H. J. Resolving Transition Metal Chemical Space: Feature Selection for Machine Learning and Structure–Property Relationships. J. Phys. Chem. A 2017, 121, 8939–8954. 10.1021/acs.jpca.7b08750. 29095620

[ref53] Janet J. P.; Gani T. Z. H.; Steeves A. H.; Ioannidis E. I.; Kulik H. J. Leveraging Cheminformatics Strategies for Inorganic Discovery: Application to Redox Potential Design. Ind. Eng. Chem. Res. 2017, 56, 4898–4910. 10.1021/acs.iecr.7b00808.

[ref54] Duan C.; Janet J. P.; Liu F.; Nandy A.; Kulik H. J. Learning from Failure: Predicting Electronic Structure Calculation Outcomes with Machine Learning Models. J. Chem. Theory Comput. 2019, 15, 2331–2345. 10.1021/acs.jctc.9b00057. 30860839

[ref55] Janet J. P.; Liu F.; Nandy A.; Duan C.; Yang T.; Lin S.; Kulik H. J. Designing in the Face of Uncertainty: Exploiting Electronic Structure and Machine Learning Models for Discovery in Inorganic Chemistry. Inorg. Chem. 2019, 58, 10592–10606. 10.1021/acs.inorgchem.9b00109. 30834738

[ref56] Janet J. P.; Duan C.; Yang T.; Nandy A.; Kulik H. J. A Quantitative Uncertainty Metric Controls Error in Neural Network-Driven Chemical Discovery. Chem. Sci. 2019, 10, 7913–7922. 10.1039/C9SC02298H. 31588334 PMC6764470

[ref57] Nandy A.; Duan C.; Janet J. P.; Gugler S.; Kulik H. J. Strategies and Software for Machine Learning Accelerated Discovery in Transition Metal Chemistry. Ind. Eng. Chem. Res. 2018, 57, 13973–13986. 10.1021/acs.iecr.8b04015.

[ref58] Janet J. P.; Ramesh S.; Duan C.; Kulik H. J. Accurate Multiobjective Design in a Space of Millions of Transition Metal Complexes with Neural-Network-Driven Efficient Global Optimization. ACS Cent. Sci. 2020, 6, 513–524. 10.1021/acscentsci.0c00026. 32342001 PMC7181321

[ref59] Glendening E. D.; Landis C. R.; Weinhold F. NBO 7.0: New vistas in localized and delocalized chemical bonding theory. J. Comput. Chem. 2019, 40, 2234–2241. 10.1002/jcc.25873. 31172571

[ref60] Gilmer J.; Schoenholz S. S.; Riley P. F.; Vinyals O.; Dahl G. E. Neural Message Passing for Quantum Chemistry. Proc. Mach. Learn. Res. 2017, 70, 1263–1272.

[ref61] Coley C. W.; Barzilay R.; Green W. H.; Jaakkola T. S.; Jensen K. F. Convolutional Embedding of Attributed Molecular Graphs for Physical Property Prediction. J. Chem. Inf. Model. 2017, 57, 1757–1772. 10.1021/acs.jcim.6b00601. 28696688

[ref62] Perdew J. P.; Burke K.; Ernzerhof M. Generalized gradient approximation made simple. Phys. Rev. Lett. 1996, 77, 3865–3868. 10.1103/PhysRevLett.77.3865. 10062328

[ref63] Weigend F.; Ahlrichs R. Balanced basis sets of split valence, triple zeta valence and quadruple zeta valence quality for H to Rn: Design and assessment of accuracy. Phys. Chem. Chem. Phys. 2005, 7, 3297 10.1039/b508541a. 16240044

[ref64] Jin W.; Barzilay R.; Jaakkola T. Junction Tree Variational Autoencoder for Molecular Graph Generation. In International Conference on Machine Learning 2018; Vol. 80, pp 2323–2332.

[ref65] Friederich P.; dos Passos Gomes G.; De Bin R.; Aspuru-Guzik A.; Balcells D. Machine Learning Dihydrogen Activation in the Chemical Space Surrounding Vaska’s complex. Chem. Sci. 2020, 11, 4584–4601. 10.1039/D0SC00445F. 33224459 PMC7659707

[ref66] Friedman J. H. Greedy Function Approximation: A Gradient Boosting Machine. Annals of Statistics 2001, 29, 1189–1232. 10.1214/aos/1013203451.

[ref67] Lu X.; Duchimaza-Heredia J.; Cui Q. Analysis of Density Functional Tight Binding with Natural Bonding Orbitals. J. Phys. Chem. A 2019, 123, 7439–7453. 10.1021/acs.jpca.9b05072. 31373822 PMC7289594

