# Supplementary material for: AABBA Graph Kernel: Atom-Atom, Bond-Bond, and Bond-Atom Autocorrelations for Machine Learning
Source: J Chem Inf Model. 2024 Nov 24;64(23):8756–69. doi: 10.1021/acs.jcim.4c01583 (PMC11632777; doi:10.1021/acs.jcim.4c01583)
Supplement: Supplementary file 1 — ci4c01583_si_001.pdf [file ci4c01583_si_001.pdf]

Supporting Information for The AABBA Graph Kernel: Atom–Atom, Bond–Bond, and Bond–Atom Autocorrelations for Machine Learning

Lucía Morán-González,[†,‡] Jørn Eirik Betten,[§] Hannes Kneiding,[†] David Balcells[†,*]

[†]*Hylleraas Centre for Quantum Molecular Sciences, Department of Chemistry, University of Oslo, P.O. Box 1033, 0315 Oslo, Norway;* [‡]*Centre for Materials Science and Nanotechnology, Department of Chemistry, University of Oslo, P.O. Box 1033, 0315 Oslo, Norway;* [§]*Simula Research Laboratory, Kristian Augusts Gate 23, 0164 Oslo, Norway*

E-mail: david.balcells@kjemi.uio.no

Table of Contents

Derivation of the AABBA autocorrelations ⋄ S3

Metal-centered depth distribution ⋄ S15

Computational details of the ML models ⋄ S16

Feature relevances from the GBM models ⋄ S21

Reduced-dimensionality Gaussian processes ⋄ S26

Reduced-dimensionality neural networks ⋄ S28

References ⋄ S30

Derivation of the AABBA autocorrelations

For a visual reference of the concepts discussed in the following sections, see Figures 1–3 in the article.

Atom–Atom Autocorrelation

The original atom–atom autocorrelation (AA-AC) of Moreau and Broto[1] transforms any connected molecular graph $\mathcal{G}$ into a fixed-length vector, v_{AA}, regardless of the size of $\mathcal{G}$. The calculation of v_{AA} is based on the autocorrelation function

$$f_{AC}(N_{\mathcal{G}}, p, d) = \sum_{i=1}^{N_{\mathcal{G}}} \sum_{j=1}^{N_{\mathcal{G}}} p_i p_j \delta_{d,d_{i,j}} \tag{S1}$$

where $N_{\mathcal{G}}$ is the number of atomic nodes in the molecular graph, p is an atomic property (*e.g.* the atomic number), i and j are atomic indices, p_i and p_j are the correlated properties of atoms i and j, and δ is the Kronecker delta; *i.e.* $\delta_{d,d_{i,j}} = 1$ for $d = d_{i,j}$ and 0 for $d \neq d_{i,j}$, where d, the depth, is the distance in the number of edges (*i.e.* chemical bonds) along the shortest path connecting the atomic nodes i and j. The f_{AC} function is permutation invariant relative to the (i, j) indices of $\mathcal{G}$.

The visual intuition behind Equation S1 is the use of the skeletal formula of a TMC as a computational graph in which the properties of the atoms are correlated by multiplication to those of the neighborhood at a given depth, adding the resulting values to obtain the components of the autocorrelation v_{AA} vector. Since, in general, the dimensionality of this vector is smaller than that of the associated graph, the $\mathcal{G} \Rightarrow v_{AA}$ transformation can be seen as a data compression operation yielding a molecular fingerprint.

The v_{AA} vector is generated by collecting the f_{AC} values at different depths, as shown in Equation S2

$$v_{AA}(D) = (f_{AC}(d = 0), f_{AC}(d = 1), ..., f_{AC}(d = D)) \tag{S2}$$

where d is expanded from $d = 0$ to the maximum depth of the representation, D, in +1 increments; *i.e.* $d \in \{0, 1, 2..., D\}$.

The v_{AA} vector is further extended by expanding the property p to a set of K atomic properties, P_A; *i.e.*

$$P_A = \{P_{A,1}, P_{A,2}, P_{A,3}..., P_{A,K}\} \tag{S3}$$

from which, considering the depth, results

$$\begin{gathered} v_{AA}(P, D) = \\ (f_{AC}(P_{A,1}, d = 0), ..., f_{AC}(P_{A,1}, d = D), ..., f_{AC}(P_{A,K}, d = 0), ..., f_{AC}(P_{A,K}, d = D)) \end{gathered} \tag{S4}$$

with an overall dimensionality of

$$dim(v_{AA}) = (D + 1) \cdot K \tag{S5}$$

For example, the use of the atomic number (Z) and covalent radius (R) as properties (*vide infra*) for a maximum depth of 3 yields the following eight-dimensional autocorrelation vector:

$$v_{AA} = (Z_0, Z_1, Z_2, Z_3, R_0, R_1, R_2, R_3) \tag{S6}$$

In addition to the depth and the atomic properties, the autocorrelation algorithm depends on two more variables; namely 1) the definition of the $d = 0$ origin, and 2) the arithmetic operator applied to the properties.

For mononuclear TMCs, the metal atom is a natural and unambiguous choice for setting the depth origin from which metal-centered (MC) autocorrelations can be computed with this equation:

$$f_{AC}(N_{\mathcal{G}}, p, d) = \sum_{j=1}^{N_{\mathcal{G}}} p_M p_j \delta_{d,d_{M,j}} \tag{S7}$$

where M is the metal center index. The other possibility is to do a full (F) autocorrelation in which all nodes are recursively used as the $d = 0$ origin once (*i.e.* Equation S1). Whereas the full AA-AC can compress more information into the v_{AA} vector, the metal-centered flavor can express electronic and steric properties over the $\{\alpha, \beta, \gamma, ...\}$ positions around the metal center, which are equivalent to $d = 0, 1, 2, ...$, in a way that organometallic and inorganic chemists can relate intuitively to proximal and distal effects.

Regarding the arithmetic operator, and besides the product autocorrelation ($\odot$), which is the one most commonly used, division, summation, and subtraction,

$$f_{AC}(N_{\mathcal{G}}, p, d) = \sum_{i=1}^{N_{\mathcal{G}}} \sum_{j=1}^{N_{\mathcal{G}}} \frac{p_i}{p_j} \delta_{d,d_{i,j}} \tag{S8}$$

$$f_{AC}(N_{\mathcal{G}}, p, d) = \sum_{i=1}^{N_{\mathcal{G}}} \sum_{j=1}^{N_{\mathcal{G}}} (p_i + p_j) \delta_{d,d_{i,j}} \tag{S9}$$

$$f_{AC}(N_{\mathcal{G}}, p, d) = \sum_{i=1}^{N_{\mathcal{G}}} \sum_{j=1}^{N_{\mathcal{G}}} (p_i - p_j) \delta_{d,d_{i,j}}, \tag{S10}$$

can also be used and referred to as ratiometric ($\oslash$; Equation S8), summetric ($\oplus$; Equation S9), and deltametric ($\ominus$; Equation S10) autocorrelations.

For TMCs, another possibility is to compute autocorrelations with distinct scopes reflecting the coordination geometry.[2] For example, for a trigonal bipyramid TMC, it is possible to define axial and equatorial terms. This option was not considered since we focused on generalizing the AABBA representations over datasets containing a wide range of different coordination geometries. Besides the Vaska's dataset,[3] in which all complexes are square planar, other datasets not used in the present study, like tmQMg,[4] also contain linear, bent, trigonal planar, tetrahedral, trigonal bipyramid, square pyramid, and octahedral TMCs.

Bond–Bond Autocorrelation

Adding to the AA-AC term, and inspired by the donor-acceptor interactions between bond orbitals in NBO analysis, we developed the bond–bond autocorrelation concept (BB-AC). In the full BB-AC implementation, bond properties are autocorrelated with the same f_{AC} function used to compute AA-AC, considering all bonds as the depth origin once. For this purpose, Equation S1 is reinterpreted as if the chemical bonds were the graph nodes; *i.e.* $N_{\mathcal{G}}$ is the number of bonds in the molecular graph, p is a bond property, i and j are bond indices, p_i and p_j are the correlated properties of bonds i and j, and, in the Kronecker delta $\delta_{d,d_{i,j}}$, d is the distance in the number of atoms along the shortest path connecting bonds i and j.

At the computational graph level, the visual interpretation of BB-AC is analogous to that of AA-AC, now feeding the product operator with bond properties instead of atom properties. As for v_{AA}, the BB-AC autocorrelation vector, v_{BB}, is composed with Equation S4 after collecting the values of its components at different depths for different properties, which can be calculated using any of the four arithmetic operators (equations S1 and S8-S10). The set of properties is

$$P_B = (P_{B,1}, P_{B,2}, P_{B,3}..., P_{B,L}) \tag{S11}$$

which contains L bond properties and, in general, $L < K$ because there are more properties available to describe the atoms than the bonds. The resulting dimensionality is thus

$$dim(v_{AC}^{BB}) = (D+1) \times L \tag{S12}$$

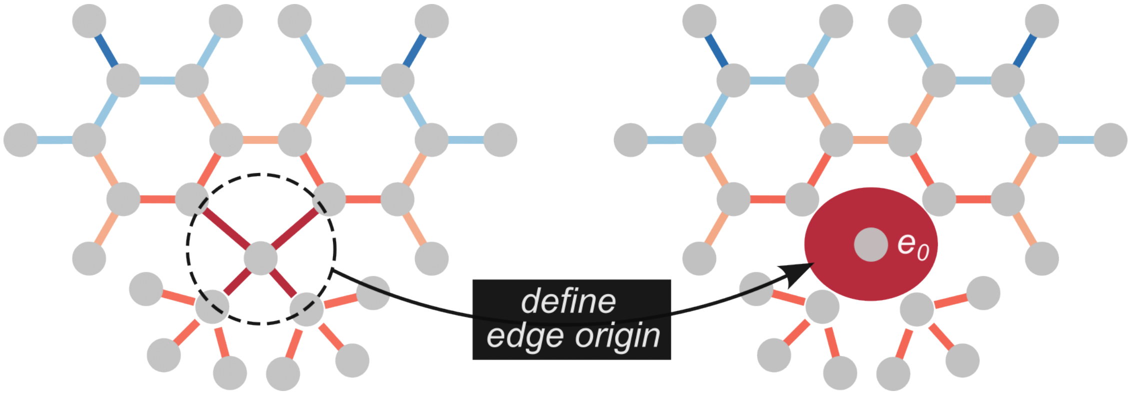

Figure S1: Definition of the super-bond edge origin e_0 (red ellipse) in the metal-centered **BB-AC** autocorrelation. The dotted-line black circle comprises the metal center and the bonds connected to it.

The metal-centered BB-AC requires redefining the depth origin. Whereas in AA-AC the origin is trivial and unique for any mononuclear TMC, in BB-AC the origin involves several bonds connecting the metal center to the ligands. Figure S1 illustrates how we implemented the BB-AC depth origin; the index of the metal center is used to identify the bonds involving it, which, as a whole, form the edge origin, e_0, at $d = 0$; *i.e.* e_0 is the following set of metal-ligand bond edges:

$$e_0 = \{e_{0,1}, e_{0,2}, ..., e_{0,CN}\} \tag{S13}$$

where CN is the coordination number of the metal center. The properties of e_0, p_{e_0}, are calculated by either summing them up:

$$p_{e_0}(CN, p) = \sum_{i=1}^{CN} p_{e_{0,i}} \tag{S14}$$

or averaging the properties of the edge set:

$$\overline{p}_{e_0}(CN, p) = \frac{1}{CN} \sum_{i=1}^{CN} p_{e_{0,i}} \tag{S15}$$

Once the edge origin and its properties are defined, the metal-centered BB-AC autocorrelations are calculated with this function:

$$f_{AC}(N_{\mathcal{G}}, p, d) = \sum_{j=1}^{N_{\mathcal{G}}} \mathcal{P}_{e_0} p_j \delta_{d,d_{e_0,j}} \tag{S16}$$

and the resulting v_{BB} vector, which collects all property and depth dimensions as in Equation S4, is labeled either $\overline{\text{BB}}$-AC or BB-AC, depending on whether $\mathcal{P}_{e_0}$ is equal to $\overline{p}_{e_0}$ or p_{e_0}, respectively.

Bond–Atom Autocorrelation

Further adding to the AA-AC and BB-AC terms, we also implemented the bond–atom autocorrelations (BA-AC), which were inspired by both NBO analysis and the coupling between embedded atom and bond properties in message-passing graph neural networks. The full BA-AC is implemented with this equation:

$$f_{AC}(N_{\mathcal{G},\mathcal{V}}, N_{\mathcal{G},\mathcal{E}}, p, d) = \sum_{i=1}^{N_{\mathcal{G},\mathcal{V}}} \sum_{j=1}^{N_{\mathcal{G},\mathcal{E}}} p_i p_j \delta_{d,d_{i,j}} \tag{S17}$$

where $N_{\mathcal{G},\mathcal{V}}$ and $N_{\mathcal{G},\mathcal{E}}$ are the number of atomic nodes and bond edges in the molecular graph, respectively, p_i and p_j are the correlated properties of atom i and bond j, and, in the Kronecker delta $\delta_{d,d_{i,j}}$, d is the distance in number of atoms between node i and bond j.

The metal-centered BA-AC is computed with this equation:

$$f_{AC}(N_{\mathcal{G},\mathcal{E}}, p, d) = \sum_{j=1}^{N_{\mathcal{G},\mathcal{E}}} p_M p_j \delta_{d,d_{M,j}} \tag{S18}$$

where p_M is an atomic property of the metal center. As for AA-AC and BB-AC, the BA-AC autocorrelation vector, v_{BA}, is composed using Equation S4 to gather all depth and property dimensions.

The product between the atomic and bond properties, which belong to sets of different dimensionality (Equations S3 and S11), was implemented as follows:

$$p_i p_j = \sum_{l=1}^{L} p_i p_{j,l} \tag{S19}$$

where, for p_j, L is the dimensionality of the bond property set and, for p_i, i is either any graph node (full BA-AC) or the metal (metal-centered BA-AC). Thus, in both cases, the final dimensionality of the representation is

$$dim(v_{BA}) = (D + 1) \times K \tag{S20}$$

where K is the dimensionality of the atomic property set.

The mixing of atomic and bond properties in Equation S17 may suggest that the term crosscorrelation could be more appropriate than autocorrelation for referring to BA-AC. However, since both terms have additional and different meanings in the field of signal processing, we decided to keep the autocorrelation term originally proposed by Moreau and Broto for molecular graphs.[1]

Atomic and bond properties

The v_{AA} vector can be derived from a set of features including atomic properties (P_A) that can be generic (*e.g.* extracted from the periodic table). In this work, we used this popular P_A set for TMCs:

$$P_A = \{Z, I, V, R, \chi\} \tag{S21}$$

From a chemical perspective, the most relevant properties are the atomic number (Z), the covalent radius (R), and the electronegativity (χ). The properties V and I are relevant from both a chemical and a graph theory perspective: V is the atomic valence, which is equal to the node degree (*i.e.* number of neighbors connected to a node), and I is the identity, which

is either 0 or 1, indicating the absence or presence, respectively, of a node at any given depth in the graph paths walked by the autocorrelation algorithm (Equation S1).

With this simple set of properties, the AA-AC autocorrelations already provide rich information about the systems they encode, including chemical composition and environment, through Z and V, and steric bulk, through R. Further, the variation of I over $d = 0, 1, ..., D$ reflects the shape of the TMC (*e.g.* linear versus branched), and, by changing the arithmetic operator, additional information can be included in the v_{AA} vector; *e.g.*, bond polarization can be encoded by applying the subtraction operator (Equation S10) to χ (*i.e.* deltametric electronegativity).

For the v_{BB} vector, we used this set of bond properties:

$$P_B = \{BO, I, BD\} \tag{S22}$$

to compute the BB-AC autocorrelations, where BO is the bond order, I is the identity, which has the same meaning as in P_A, and BD is the bond distance in Å, which gives further geometric information in addition to that provided by R and I in P_A.

Except for the bond distance, the properties included in the P_A and P_B sets are generic, thus having a limited capacity in distinguishing different chemical environments; *e.g.* the C atom of any R–CH_2–R' fragment is described with the same P_A values regardless of the nature of R and R'. This limitation can be tackled by using specific electronic structure properties from inexpensive quantum mechanical calculations. In a recent study, we showed that the leverage of this electronic structure information in graph neural networks boosts the prediction accuracy of the resulting models to an extent larger than that provided by geometric information.[4] In the present work, we investigated the use of NBO data in the computation of the autocorrelation vectors.

Table S1: NBO properties included in the atomic ($P_{A,NBO}$) and bond ($P_{B,NBO}$) property sets.[a]

	$P_{A,NBO}$		$P_{B,NBO}$
Z	Atomic number	BD	Bond distance (Å)
q_{Nat}	Natural charge (e)	BO_{Nat}	Natural Wiberg bond order
V_{Nat}	Natural valence index	N_{BN}	# bonding NBOs
N_s	# s electrons in nat. config.	BN_E	E of highest-lying BN (Ha)
N_p	# p electrons in nat. config.	$BN_{\Delta E}$	Lowest/highest-lying BN E gap (Ha)
N_d	# d electrons in nat. config.	BN_{Occ}	Electron occupancy of highest-E BN
N_{LP}	# Lone pairs	BN_s	s-character of highest-E BN (%)
LP_E	E of highest-lying LP (Ha)	BN_p	p-character of highest-E BN (%)
$LP_{\Delta E}$	Lowest/highest-lying LP E gap (Ha)	BN_d	d-character of highest-E BN (%)
LP_{Occ}	Electron occupancy of highest-E LP	N_{BN^*}	# non- & anti-bonding NBOs
LP_s	s-character of highest-E LP (%)	BN^*_E	E of lowest-lying BN* (Ha)
LP_p	p-character of highest-E LP (%)	$BN^*_{\Delta E}$	Lowest/highest-lying BN* E gap (Ha)
LP_d	d-character of highest-E LP (%)	BN^*_{Occ}	Electron occupancy of lowest-E BN*
N_{LV}	# Lone vacancies	BN^*_s	s-character of lowest-E BN* (%)
LV_E	E of lowest-lying LV (Ha)	BN^*_p	p-character of lowest-E BN* (%)
$LV_{\Delta E}$	Lowest/highest-lying LV E gap LV (Ha)	BN^*_d	d-character of lowest-E BN* (%)
LV_{Occ}	Electron occupancy of lowest-E LV		
LV_s	s-character of lowest-E LV (%)		
LV_p	p-character of lowest-E LV (%)		
LV_d	d-character of lowest-E L (%)		

[a]Abbreviations: # = Number of; E = Energy; Nat. = Natural; LP = Lone Pair; LV = Lone Vacancy; Config. = Configuration; BO = Bond Order; NBOs = Natural Bond Orbitals; BN = Bonding NBO; BN* = Non- and anti-bonding NBOs.

The set of NBO atomic properties, $P_{A,NBO}$, includes the atomic number, the natural charge and valence index, the number of lone pairs (LP) and vacancies (LV), and the electron occupancies and symmetries of the highest- and lowest-energy LP and LV orbitals, respectively. The set of NBO bond properties, $P_{B,NBO}$, includes the same information for the bonding and antibonding valence orbitals, as well as the natural bond order and the bond distance. Table S1 provides a systematic list of all NBO data included in $P_{A,NBO}$ and $P_{B,NBO}$.

Whole-graph properties

We defined a set of whole-graph properties; *i.e.*

$$P_{\mathcal{G}} = \{q, M, N_{At}, N_e\}, \tag{S23}$$

which contains the charge of the metal complex (q), its molecular mass (M), and the total number of atoms (N_{At}) and electrons (N_e). These properties were appended to the end of the AABBA autocorrelation vector in the ML models; *i.e.*

$$v^{\mathcal{G}}_{AABBA} = v_{AABBA} \frown P_{\mathcal{G}} \tag{S24}$$

where v_{AABBA} is either v^{I}_{AABBA} or v^{II}_{AABBA}, as described in the next section.

Atom–Atom Bond–Bond Bond–Atom Autocorrelations

With the aim of developing a molecular graph-to-vector transformation in which both atom and bond properties are autocorrelated separately and jointly, we developed an atom–atom, bond–bond, bond–atom AABBA graph kernel yielding v_{AABBA} autocorrelation vectors through two distinct implementations that can be regarded as being either explicit or implicit.

In the explicit implementation of the graph kernel, AABBA(I), the resulting vector representation, v^{I}_{AABBA}, was composed by simply joining the AA-AC, $\overline{\mathrm{BB}}$-AC, and BA-AC autocorrelations as follows:

$$v^{I}_{AABBA} = v_{AA} \oplus v_{\overline{BB}} \oplus v_{BA} \tag{S25}$$

which has dimensionality

$$dim(v^I_{AABBA}) = (D+1) \times (2K+L) \tag{S26}$$

where D = maximum depth, $K = dim(P_A)$, and $L = dim(P_B)$; the NBO $P_{A,NBO}$ and $P_{B,NBO}$ property sets can be also correlated, expanding the dimensionality of the resulting representations.

In the implicit implementation, AABBA(II), the v^{II}_{AABBA} vector was computed with the bond–bond autocorrelation function, as defined in Equation S16, applied to property sets that describe both the bond and the atoms associated to it (P_{AB}); in particular, for any i–j bond edge connecting the atomic nodes i and j, we considered these three sets based on generic properties:

$$\begin{aligned} P_{AB,1} &= \{Z_i, Z_j, V_i, V_j, \chi_i, \chi_j, BD, BO, I\}; M = 9 \\ P_{AB,2} &= \{Z_i, Z_j, V_i, V_j, \chi_i - \chi_j, BD, BO, I\}; M = 8 \\ P_{AB,3} &= \{Z_i, Z_j, V_i, V_j, \chi_i - \chi_j, R_i, R_j, BO, I\}; M = 9 \end{aligned} \tag{S27}$$

In $P_{AB,1}$, each bond is described by its distance and order, whereas the associated atoms are described by their atomic number, valence, and electronegativity. In $P_{AB,2}$, the latter is replaced by the $\chi_i - \chi_j$ difference, which accounts for the polarization of the i–j bond. Lastly, in $P_{AB,3}$, the bond distance is replaced by the covalent radii of the atoms to yield a geometry-agnostic representation. We also defined two additional P_{AB} sets based on NBO properties:

$$\begin{gathered} P_{AB,4} = \{q_{Nat,i}, q_{Nat,j}, V_{Nat,i}, V_{Nat,j}, N_{s,i}, N_{s,j}, N_{p,i}, N_{p,j}, N_{d,i}, N_{d,j}, N_{LP,i}, N_{LP,j}, N_{LV,i}, N_{LV,j}, \\ BD, BO_{Nat}, N_{BN}, BN_s, BN_p, BN_d, N_{BN^*}, BN^*_s, BN^*_p, BN^*_d, I\}; M = 25 \end{gathered} \tag{S28}$$

$$P_{AB,5} = \{q_{Nat,i}, q_{Nat,j}, V_{Nat,i}, V_{Nat,j}, N_{LP,i}, N_{LP,j}, LP_{E,i}, LP_{E,j}, LP_{\Delta E,i}, LP_{\Delta E,j},$$
$$N_{LV,i}, N_{LV,j}, LV_{E,i}, LV_{E,j}, LV_{\Delta E,i}, LV_{\Delta E,j}, BD, BO_{Nat}, N_{BN}, BN_E, BN_{\Delta E}, \quad \text{(S29)}$$
$$N_{BN^*}, BN^*_E, BN^*_{\Delta E}, I\}; M = 25$$

where $P_{AB,4}$ is rich in orbital symmetry information whereas $P_{AB,5}$ is rich in orbital energy information.

The resulting autocorrelations were labeled AABBA(II)$_n$, where n is the index of the $P_{AB,n}$ property set used in their calculation. The dimensionality of the associated vectors is

$$dim(v^{II}_{AABBA}) = (D + 1) \times M \quad \text{(S30)}$$

where M is the number of properties included in the P_{AB} sets, as shown in Equations S27, S28 and S29. Both the AABBA(I) and AABBA(II)$_n$ kernels are available in the full and metal-centered flavors.

Maximal dimensionality autocorrelation vectors

With the aim of selecting and interpreting features with GBM models, we extended the autocorrelation vectors to maximal dimensionality (MD). For both the generic and NBO properties, separately, we used the AABBA(I) graph kernel to compute the $v^{I,MD}_{AABBA}$ autocorrelation vectors, with the concatenation operation defined in Equation S25, and including both the BB-AC and $\overline{\text{BB}}$-AC autocorrelations in the bond–bond term. Further, all terms were expanded in both full and metal-centered fashions, and using, in this order, the product, subtraction, division, and summation operators; for example, for the atom–atom autocorrelation:

$$v_{AA} \in v^{I,MD}_{AABBA} = (v^{\odot}_{AA} \oplus v^{\ominus}_{AA} \oplus v^{\oslash}_{AA} \oplus v^{\oplus}_{AA})_{full} \ \oplus \ (v^{\odot}_{AA} \oplus v^{\ominus}_{AA} \oplus v^{\oslash}_{AA} \oplus v^{\oplus}_{AA})_{MC} \tag{S31}$$

in which each vector component was expanded from depth zero to six (Figure S1).

Metal-centered depth distribution

The histograms in Figure S2 show the distribution of maximal metal-centered depth values derived from two different walks: through the nodes (top plot) and through the edges (bottom plot) over the Vaska's dataset. The walk through the nodes is depicted in Figure S3.The extreme value of 6 for the maximal metal-centered depth in the node walk was taken as the limit in both the metal-centered and full autocorrelations.

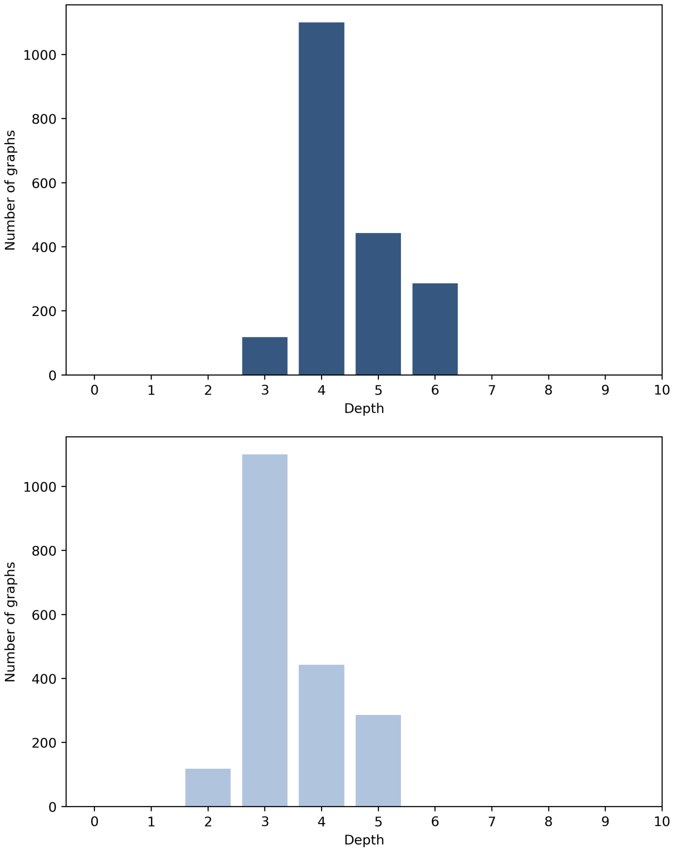

Figure S2: Maximal metal-centered depths distribution walking through the nodes (top) and through the edges (bottom) over the Vaska's dataset.

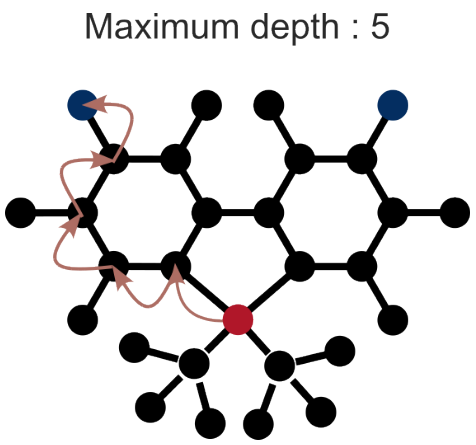

Figure S3: The maximal metal-centered depth is defined as the length of the shortest path connecting the metal center to the furthest atomic node, here illustrated for a random TMC graph.

Computational details of the ML models

Neural networks

The neural network models were based on a multilayer perceptron architecture with the following hyperparameters: two hidden layers, 128 nodes per layer, ReLU activation, Adam optimizer, and a training:validation:test data split of 80:10:10. All models were trained using a batch size of 32 for 200 epochs. The learning rate (lr) was dynamic; *i.e.* after lr = 0.01 initialization, it was monitored with the `ReduceLROnPlateau` scheduler of the *PyTorch* library[5] using a factor of 0.7 to scale the lr whenever the validation error did not decrease for five epochs. For each optimization, the model yielding the highest validation accuracy was reassessed over the test dataset, giving the error metrics reported in the article.

To evaluate the robustness of the best ML models in the prediction of both the Vaska's energy barrier (model 13 in Table S2) and breaking H$\cdots$H distance (model 18 in Table S3), we repeated ten times the models training on a different random training:validation:test split to further confirm their performance.

Table S2: Average and lowest test errors in the prediction of the Vaska's dataset energy barriers with neural networks. The inputs passed to the models were vectors defined with different graph kernels ($\mathcal{G}_{\mathcal{K}}$), property types (Prop), operators ($\hat{\text{O}}$p), origins (Ø), and maximum depths (D), yielding different dimensionality (*dim*). The mean absolute error (MAE) is given in kcal/mol.

Model	Input						Average Error		Lowest Error	
	$\mathcal{G}_{\mathcal{K}}$	Prop	$\hat{\text{O}}$p	Ø	D	*dim*	MAE	r^2	MAE	r^2
13	I	NBO	$\odot$	F	3	223	0.79 ± 0.04	0.927 ± 0.001	0.70	0.945

Table S3: Average and lowest test errors in the prediction of the Vaska's dataset H···H distance with neural networks. The inputs passed to the models were vectors defined with different graph kernels ($\mathcal{G}_{\mathcal{K}}$), property types (Prop), operators ($\hat{\text{O}}$p), origins (Ø), and maximum depths (D), yielding different dimensionality (*dim*). The mean absolute error (MAE) is given in Å.

Model	Input						Average Error		Lowest Error	
	$\mathcal{G}_{\mathcal{K}}$	Prop	$\hat{\text{O}}$p	Ø	D	*dim*	MAE	r^2	MAE	r^2
18	II_3	P	$\odot$	MC	3	33	$1.88{\cdot}10^{-2} \pm 8{\cdot}10^{-4}$	0.721 ± 0.036	$1.76{\cdot}10^{-2}$	0.769

Gradient boosting machines

The ensemble model chosen is a Gradient Boosting Machine (GBM) model,[6,7] an ensemble of simple estimators (regression trees in this case) fitted in a forward-stagewise fashion to the residual error on the training set. The regression trees split the domain into regions, returning the average output within the region from the training set as the estimate of the predicted output.

The GBM model was implemented using the `GradientBoostingRegressor` from the `scikit-learn`-library[8] and the hyperparameters chosen for the GBM implementations are given in Table S4. The loss function measures the fit, which, in this case, is calculated as the squared error. The learning rate hyperparameter multiplies the contribution of each regression tree added to the model with its value, and a low value here will make it possible to add more regression trees to the model before overfitting occurs. The boosting iterations refer to the number of regression trees added to the model, while the maximal depth refers to the maximal depth of each regression tree in the model. The choice of setting the maximal depth of the regression trees to 5 is done because the individual regression trees will have low variance, but can still make regions defined by up to five autocorrelation descriptors, which should catch many of the dependencies of the output given the input. The rest of the

hyperparameters were set to the default values defined for the `scikit-learn` library.

Table S4: Hyperparameters chosen for the GBM models.

Hyperparameter	Value
Loss	Squared error
Learning rate	0.05
Boosting iterations	1000
Maximal depth	5

The GBM model was evaluated using 5-fold cross-validation on a randomly shuffled dataset. This would correspond to five different training and test sets, each of them with a size ratio of 80:20. The reported evaluation measure is the mean absolute error, and the models were evaluated against the mean absolute error in addition to the mean squared error. The models chosen were the best performing models on the test set, which were the full models of 1000 regression trees. This means that every addition of a simple estimator to the model in the forward–stagewise process resulted in a reduction of the test error, not only the training error, indicating no signs of overfitting.

Feature relevance was calculated using the Friedman MSE criterion (i^2) for all associated splits:[6]

$$i^2(R_l, R_r) = \frac{n_l n_r}{n_l + n_r}(\bar{y}_l - \bar{y}_r)^2 \tag{S32}$$

where R_l and R_r correspond to the left and right regions of a split, respectively, and n_l is the number of data points in the training set within the left region, $\bar{y}_l$ the mean target response of these data points, n_r denotes the number of data points in the training set within the right region, and $\bar{y}_r$ the corresponding mean target response.

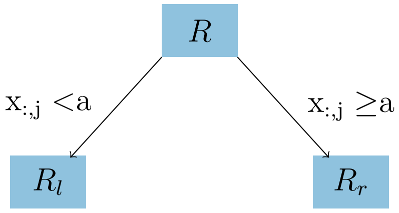

Figure S4: Visualization of a split of a region R into two regions R_l and R_r.

We can intuitively think of the Friedman criterion as the reduction in the residual sum of squares (RSS) after the addition of the split to the model. More specifically, given that an input space R, which contains n data points, is split into two regions, R_l and R_r, based on the value of a feature $\mathrm{x}_{:,\mathrm{j}}$ (*i.e.* the jth feature of all data points; Figure S4), the predicted value for the region R (before the split) is $\hat{y} = \frac{1}{n}\sum_{x_i \in R}^{n} y_i$, and we denote the residual sum of squares of the region R as

$$\mathrm{RSS}(R) = \sum_{i=1}^{n}(y_i - \hat{y})^2$$

and recalling that the regions R_l and R_r are sub-regions of R, such that $R_l + R_r = R$, we can adopt the notation

$$\sum_{i=1}^{n} y_i \mathbf{1}(x_i \in R_l) = \sum_{i_l=1}^{n_l} y_{i_l}$$
$$\sum_{i=1}^{n} y_i \mathbf{1}(x_i \in R_r) = \sum_{i_r=1}^{n_r} y_{i_r}$$

and considering that

$$\sum_{i=1}^{n} y_i = \sum_{i_l=1}^{n_l} y_{i_l} + \sum_{i_r=1}^{n_r} y_{i_r}$$

the model predictions in the sub-regions R_r and R_l are

$$\hat{y}_r = \frac{1}{n_r}\sum_{i_r=1}^{n_r} y_{i_r}$$

and

$$\hat{y}_l = \frac{1}{n_l}\sum_{i_l=1}^{n_l} y_{i_l}$$

and, therefore, the improvement in the residual sum of squares before and after the split, denoted ΔRSS, can be defined as

$$\Delta\text{RSS} = \text{RSS}(R) - \text{RSS}(R_r) - \text{RSS}(R_l)$$

Finally, considering that

$$\hat{y} = \frac{1}{n}\sum_{i}^{n} y_i = \frac{n_r n_l}{n_r + n_l}\left(\frac{\hat{y}_r}{n_l} + \frac{\hat{y}_l}{n_r}\right)$$

we can reformulate the ΔRSS expression as

$$\Delta\text{RSS} = \frac{n_r n_l}{n_r + n_l}(\hat{y}_r - \hat{y}_l)^2$$

which is exactly Equation S32.

The Friedman MSE is therefore a measure for the improvement in the residual sum of squares. Using it, relevance is calculated for each feature by summing all Friedman MSE contributions from each corresponding split and dividing the result by the total sum of all Friedman MSE contributions over the entire gradient boosting model. If we denote all splits in the model by the set $S = \{(R_1, R_2), (R_3, R_4), \ldots, (R_{L-1}, R_L)\}$, and the subset of splits using feature $x_{:,k}$ as $S_k \subseteq S$, we can formally define the relevance as

$$\text{relevance}(x_{:,k}) = \frac{\sum_{(R_i, R_{i+1}) \in S_k} i^2(R_i, R_{i+1})}{\sum_{(R_j, R_{j+1}) \in S} i^2(R_j, R_{j+1})}$$

Gaussian processes

Gaussian processes were implemented using the `GPyTorch`-library[9] and the Linear-RBF kernel described in the manuscript. The mean function chosen was the constant mean function. We applied the `FixedNoiseGaussianLikelihood` from the `GPyTorch`-library as the likelihood function, which has a parameter that only fixes the lower bound of the noise, assuming the standard homoskedastic model; *i.e.*

$$\Pr(Y = y|f) = f + \mathcal{N}(0, \epsilon^2) \tag{S33}$$

The GP models were trained with the same 80:10:10 split, choosing the model that, over 100 training epochs, minimized the validation error. The criterion used for training was to maximize the marginal log likelihood of the training data. The parameters that were updated within the model were the variance, σ^2, the length scale, λ, and the noise parameter, ϵ, of the likelihood function (Equation S33). The learning rate was initially set to 1 and thereafter updated with the `ReduceLROnPlateau` scheduler of the `PyTorch`-library,[5] using a factor of 0.7. The lower bound on the noise parameter of the likelihood was set to 10^{-4}.

Feature relevances from the GBM models

From a chemical perspective, the type of autocorrelation features found relevant in the prediction of the Vaska's activation barrier and breaking H$\cdots$H distance may give further insight into the nature of the reaction. Figures S5, S6 and S7 show the 20 most relevant autocorrelation features as calculated by the GBMs, where only atom–atom, bond–bond, and bond–atom autocorrelation features where used as input, respectively. These results were produced using 5-fold cross-validation on the entire dataset.

For the generic property sets using only atom-atom autocorrelation features (Figure S5), we observe that the features based on the chemical composition of the iridium complex play a crucial role (Z) in the prediction of the activation barrier, together with the summetric

polarity of the first coordination sphere of the metal center (χ-1-MD), and a ratiometric of the electronegativity between the metal and the first coordination sphere (χ-1-MR). Similarly, for the NBO properties, we observe that the number of d-electrons in the natural configuration (N_d-0-FA) of the entire complex is the single most important feature for the prediction of the activation barrier. Furthermore, we note that the lone pair and natural charge properties are important in the prediction of the barrier, and there are more full autocorrelations than metal-centered.

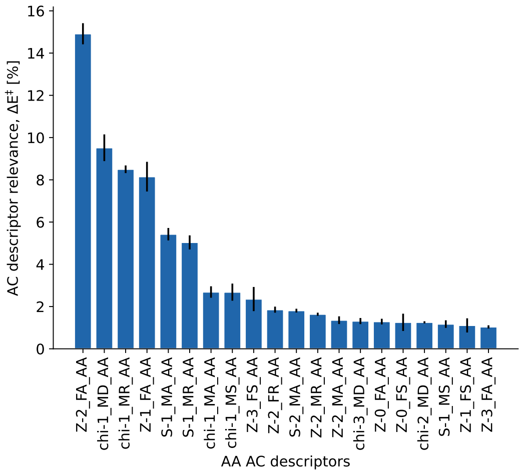

(a) Top 20 generic atom-atom AC descriptors for barrier prediction.

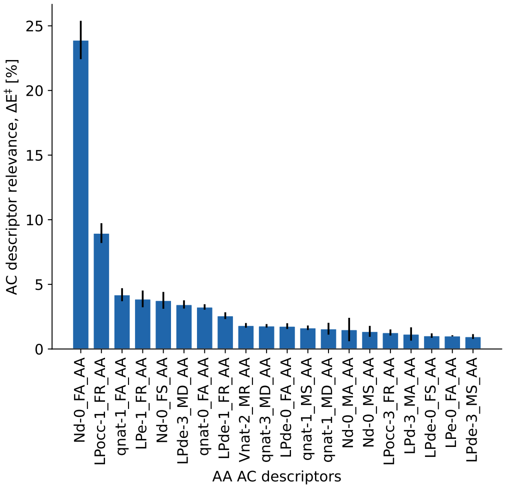

(b) Top 20 NBO atom-atom AC descriptors for barrier prediction.

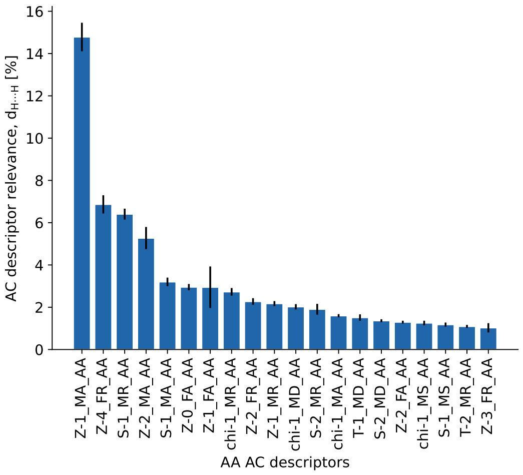

(c) Top 20 generic atom-atom AC descriptors for H···H distance prediction.

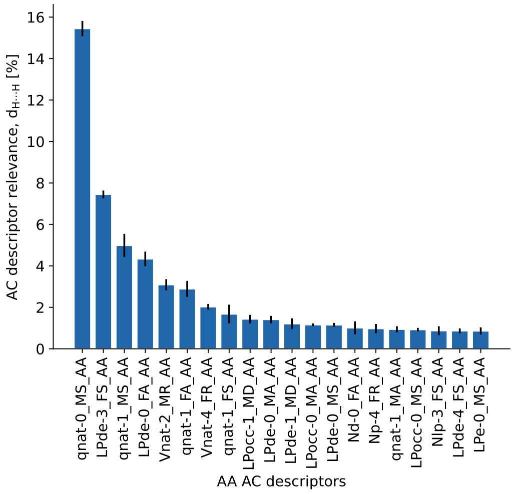

(d) Top 20 NBO atom-atom AC descriptors for H···H distance prediction.

Figure S5: Relevance of atom–atom autocorrelation descriptors.

In the prediction of $d_{H\cdots H}$, we count more metal-centered autocorrelation features among the most important, in line with the breaking of the H$\cdots$H-bond taking place within the first coordination sphere. We also observe that autocorrelation features based on atom number and electronegativity (Z and χ) are important using the generic properties, but we also observe that the ratio and product of the size of the first coordination sphere and the metal are relevant. The NBO-based autocorrelation features for this prediction task are metal-centered and focus on natural charge, lone pairs, and natural valence.

In the prediction of the barrier using only bond-bond autocorrelation features (Figure S6), we observe that the difference in bond order between the first coordination sphere and second coordination sphere with the metal super-bond are the two most important features. Next, there are measures of the average distances of the bonds to the metal (d-0 terms). Almost all the features among the 20 most relevant are highly local around the metal center. Likewise, for the NBO-calculated autocorrelation features, we observe that features based on the occupancies of the valence NBOs are important, together with bond orders and distance terms, which are mostly focused on the metal center.

The H$\cdots$H distance prediction uses features with bond distance autocorrelations over the first and second coordination spheres, with the metal super-bond, as well as properties of the metal super-bond, to a large extent. Bond orders seem to be less important in this prediction task. Similarly, when using the NBO properties, we observe that bond distance autocorrelation properties of the metal super-bond are highly correlated with the target.

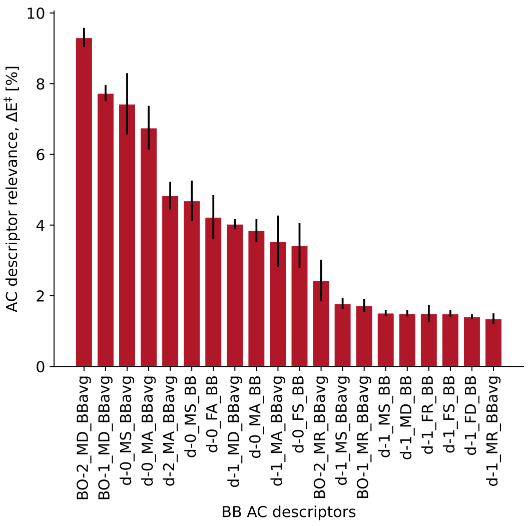

(a) Top 20 generic bond-bond AC descriptors for barrier prediction.

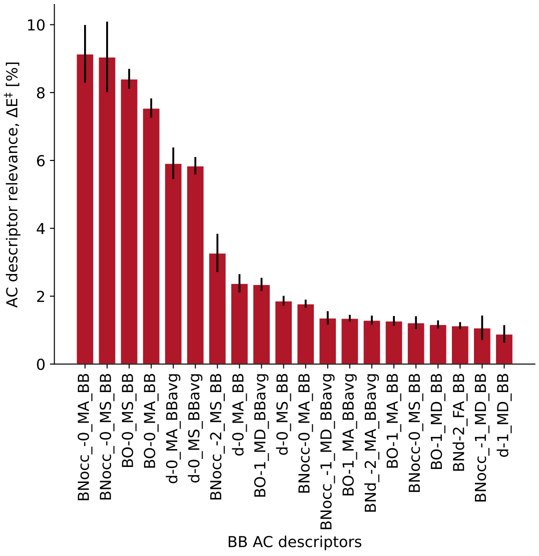

(b) Top 20 NBO bond-bond AC descriptors for barrier prediction.

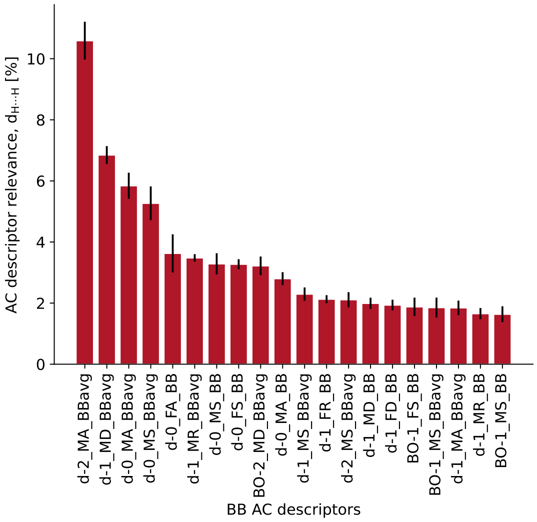

(c) Top 20 generic bond-bond AC descriptors for H$\cdots$H distance prediction.

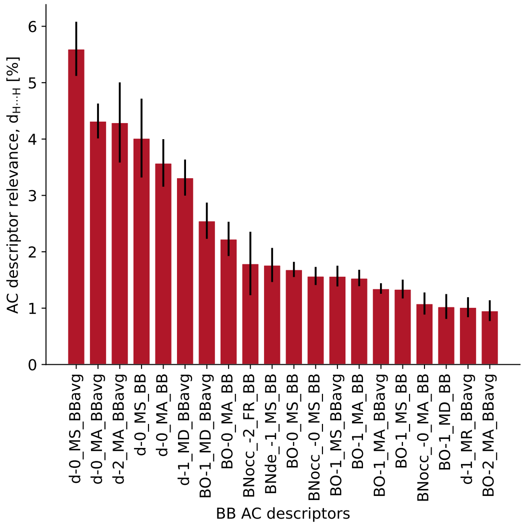

(d) Top 20 NBO bond-bond AC descriptors for H$\cdots$H distance prediction.

Figure S6: Relevance of bond–bond autocorrelation descriptors.

Regarding Figure S7, it is worth noting that, since the bond–atom autocorrelation terms are atom properties correlated with bond properties, the zero-depth metal-centered terms are indeed autocorrelating the metal atom properties to those of the metal–ligand bonds. The prediction of $\Delta E^{\ddagger}$ is strongly correlated with the difference between the size of the metal center and all the bond properties of the super-bond (S-0-MD), while the I-1-MD and I-2-MD terms can be thought of as a sum counting all bond properties in the first

and second coordination spheres of the metal. Properties using the electronegativity and atomic numbers are also important in this case. Almost every autocorrelation feature highly correlated with the target is metal-centered, which is also observed when using the NBO properties. More specifically, in the latter case, we observe that the autocorrelation between the natural charge of the metal center with bonds between the zeroth and first, first and second, and second and third coordination spheres of the metal are the most important features for the prediction of the barrier. Furthermore, we note more features using the lone pair and natural charge of the metal center, with a few of them being full autocorrelation features.

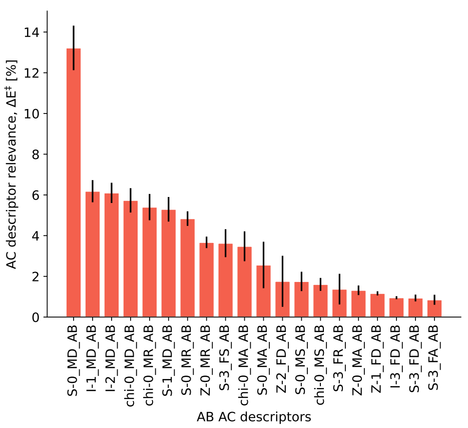

(a) Top 20 generic bond-atom AC descriptors for barrier prediction.

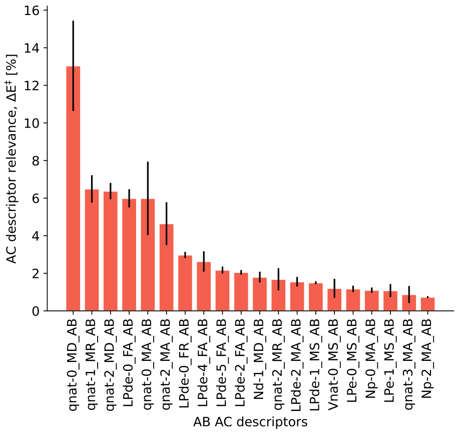

(b) Top 20 NBO bond-atom AC descriptors for barrier prediction.

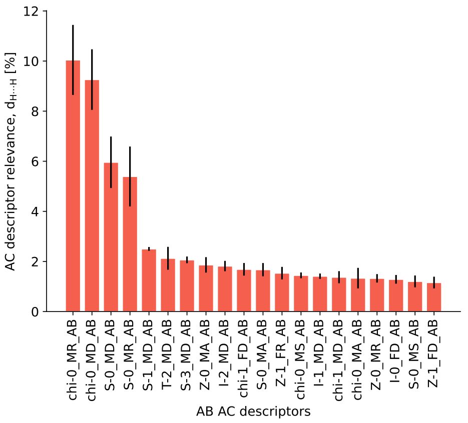

(c) Top 20 generic bond-atom AC descriptors for H· · · H distance prediction.

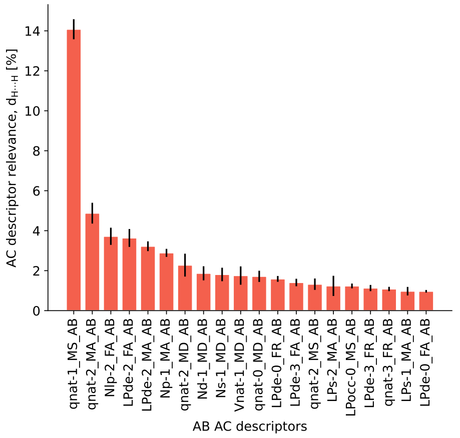

(d) Top 20 NBO bond-atom AC descriptors for H· · · H distance prediction.

Figure S7: Relevance of bond-atom autocorrelation descriptors.

Reduced-dimensionality Gaussian processes

Tables S5 and S6 show performance metric values in the 50-100% range of accumulated relevance in 5% increments for the prediction of the Vaska's energy barriers and breaking H···H distances, respectively, using Gaussian processes. Accumulated relevances are those found with the GBM models and, in both tables, the last entry corresponds to the full autocorrelation vector.

Table S5: Test errors in the prediction of the Vaska's dataset energy barrier ($\Delta E^{\ddagger}$) with Gaussian processes. The inputs passed to the models were $v^{I,MD}_{AABBA}$ vectors defined with property types Prop after being clipped to a reduced dimensionality (*rdim*) where the remaining relevance left in the vector, as calculated by the GBM, is given under AR (accumulated relevance). The mean absolute errors (MAEs) are given in kcal/mol.

Target	Input (Prop=P[a])			Error[b]		Input (Prop=NBO[c])			Error[b]	
	rdim[d]	AA:BB:BA	AR	MAE	r^2	*rdim*[d]	AA:BB:BA	AR	MAE	r^2
$\Delta E^{\ddagger}$	11	5:4:2	50%	1.28	0.830	12	4:2:6	50%	1.16	0.845
$\Delta E^{\ddagger}$	14	6:5:3	55%	1.28	0.820	16	5:5:6	55%	1.11	0.848
$\Delta E^{\ddagger}$	17	7:7:3	60%	1.26	0.837	21	9:6:6	60%	0.91	0.868
$\Delta E^{\ddagger}$	21	7:8:6	65%	1.23	0.829	27	12:9:6	65%	0.88	0.887
$\Delta E^{\ddagger}$	27	8:9:10	70%	0.93	0.8941	35	16:11:8	70%	0.87	0.860
$\Delta E^{\ddagger}$	35	14:9:12	75%	0.77	0.933	47	23:15:9	75%	0.94	0.849
$\Delta E^{\ddagger}$	46	19:12:15	80%	0.67	0.947	68	34:18:16	80%	0.84	0.901
$\Delta E^{\ddagger}$	63	30:15:18	85%	0.69	0.945	115	53:29:33	85%	0.79	0.927
$\Delta E^{\ddagger}$	93	44:20:29	90%	0.72	0.939	177	70:53:54	90%	0.79	0.925
$\Delta E^{\ddagger}$	161	63:34:64	95%	0.72	0.935	498	172:174:152	95%	0.80	0.914
$\Delta E^{\ddagger}$	532	163:125:244	100%	0.86	0.914	1916	433:773:710	100%	0.99	0.873
$\Delta E^{\ddagger}$	671	223:188:260	100%	0.90	0.904	2750	680:1068:1002	100%	0.94	0.873

[a]*I.e.* P_A, P_B, and P_{AB} generic property sets; [b]From training the GP on 80% of the dataset; [c]*I.e.* $P_{A,NBO}$, $P_{B,NBO}$, and $P_{AB,NBO}$ NBO property sets; [d]After clipping the AC vector with respect to relevance. See Appendix for further details.

In the prediction of the barrier with the generic property set, we observe that an accumulated relevance of around 80%, which reduces the input from the original 671 autocorrelation features to 46 has the highest performance, lowering the MAE from 0.90 kcal/mol (full vector) to 0.67 kcal/mol (reduced vector). Similarly, when using NBO properties, we observe that the MAE minimum is at ∼85% accumulated relevance, corresponding to an input vector of size 115, reduced down from the 2750 dimensions of the original full vector. In this case, the MAE was lowered from 0.94 to 0.79 kcal/mol.

Table S6: Test errors in the prediction of the Vaska's dataset H···H distance ($d_{H\cdots H}$) with Gaussian processes. The inputs passed to the models were $v^{I,MD}_{AABBA}$ vectors defined with different property types (Prop) after being clipped to a reduced dimensionality (*rdim*) where the remaining relevance left in the vector, as calculated by the GBM, is given under AR (accumulated relevance). The mean absolute errors (MAEs) are given in Å for the distances.

Target	Input (Prop=P[a])			Error[b]		Input (Prop=NBO[c])			Error[b]	
	rdim[b]	AA:BB:BA	AR	MAE	r^2	*rdim*[d]	AA:BB:BA	AR	MAE	r^2
$d_{H\cdots H}$	15	7:4:4	50%	2.00·10^{-2}	0.681	24	11:7:6	50%	1.79·10^{-2}	0.765
$d_{H\cdots H}$	20	10:5:5	55%	1.79·10^{-2}	0.792	35	18:8:9	55%	1.77·10^{-2}	0.775
$d_{H\cdots H}$	26	12:7:7	60%	1.75·10^{-2}	0.799	52	24:12:16	60%	1.84·10^{-2}	0.752
$d_{H\cdots H}$	34	16:9:9	65%	1.65·10^{-2}	0.832	85	38:23:24	65%	1.88·10^{-2}	0.755
$d_{H\cdots H}$	46	20:12:14	70%	1.65·10^{-2}	0.832	115	48:33:34	70%	2.01·10^{-2}	0.696
$d_{H\cdots H}$	63	24:17:22	75%	1.68·10^{-2}	0.829	163	69:49:45	75%	1.99·10^{-2}	0.706
$d_{H\cdots H}$	90	36:22:32	80%	1.63·10^{-2}	0.833	208	82:72:56	80%	1.93·10^{-2}	0.753
$d_{H\cdots H}$	120	49:26:45	85%	1.71·10^{-2}	0.799	296	127:114:100	85%	1.98·10^{-2}	0.736
$d_{H\cdots H}$	169	65:33:71	90%	1.84·10^{-2}	0.777	429	153:152:127	90%	1.99·10^{-2}	0.731
$d_{H\cdots H}$	244	93:54:127	95%	1.73·10^{-2}	0.811	657	238:318:262	95%	4.52·10^{-2}	-0.118
$d_{H\cdots H}$	510	160:112:238	100%	1.88·10^{-2}	0.770	1971	436:789:746	100%	4.42·10^{-2}	-0.072
$d_{H\cdots H}$	671	223:188:260	100%	1.93·10^{-2}	0.766	2750	680:1068:1002	100%	4.50·10^{-2}	-0.103

[a] *I.e.* P_A, P_B, and P_{AB} generic property sets; [b] From training the GP on 80% of the dataset; [c] *I.e.* $P_{A,NBO}$, $P_{B,NBO}$, and $P_{AB,NBO}$ NBO property sets; [d] After clipping the AC vector with respect to relevance. See Appendix for further details.

In the prediction of the breaking H···H distances, the MAE is minimized at ∼80% accumulated relevance, using the generic property sets. This reduces the dimensionality of the input from 671 to 90 features and the corresponding MAE from 1.93·10^{-2} to 1.63·10^{-2} Å. With the NBO properties, the minimal MAE is at ∼55% accumulated relevance, corresponding to a 35-dimensional input vector, down from the original 2750 dimensions, and reducing the MAE to 1.77·10^{-2} Å. Interestingly, this GP model could not be optimized for this regression task using the full-dimension vector, which yielded a MAE of 4.50·10^{-2}, with $r^2 = -0.103$.

The MAE of the barrier prediction as a function of accumulated relevance is shown for the NBO properties in Figure S8. This figure highlights the multiple local minima of the function at approximately 67% and 86% accumulated relevances. An important implication of this non-convex nature is that an algorithm that searches over this space cannot simply stop when the estimated derivative changes sign one time but instead has to complete a more complex exploration.

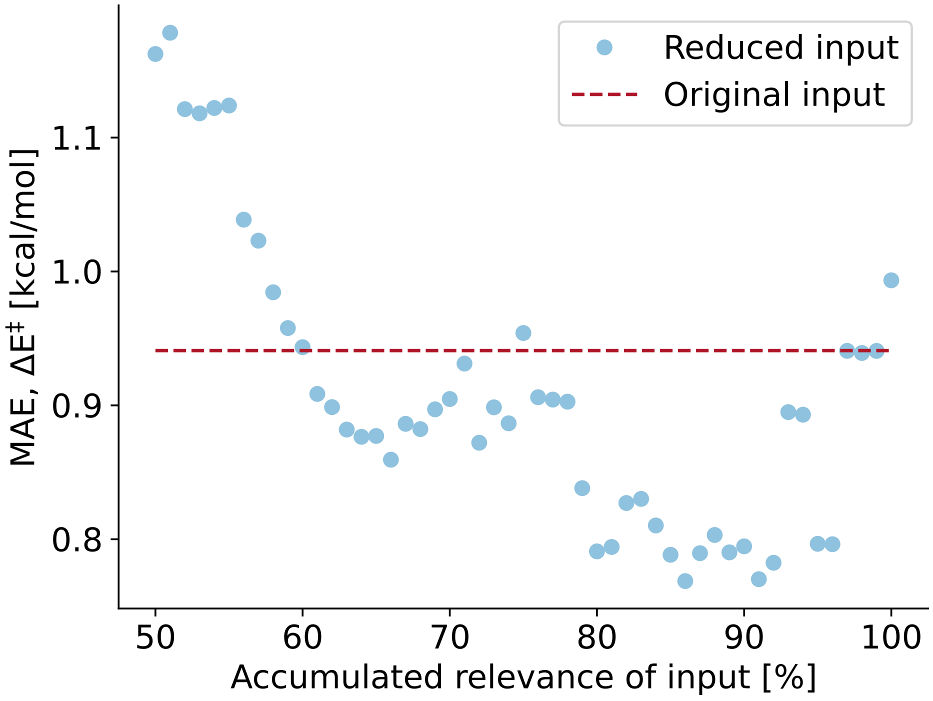

Figure S8: Influence of reducing the dimensionality of the AABBA representation in the MAEs of the GP models predicting the Vaska's energy barriers ($\Delta E^{\ddagger}$; top) from NBO properties.

Reduced-dimensionality neural networks

For the same accumulated relevances of Tables S5 and S6, the results of the NN models are shown in Figure S9 at a lower resolution, in terms of the dimensionality of the input vectors. Similarly to the GP experiments, we observe a minima for the prediction of $\Delta E^{\ddagger}$ with the dimensionality of the input at 46 for the generic properties, and at a dimensionality of 177 for the NBO (115 with Gaussian processes). In the prediction of $d_{H\cdots H}$, the minima are located at dimensionalities 90 and 35 for the generic and NBO property sets, respectively, exactly the same as for the GP models.

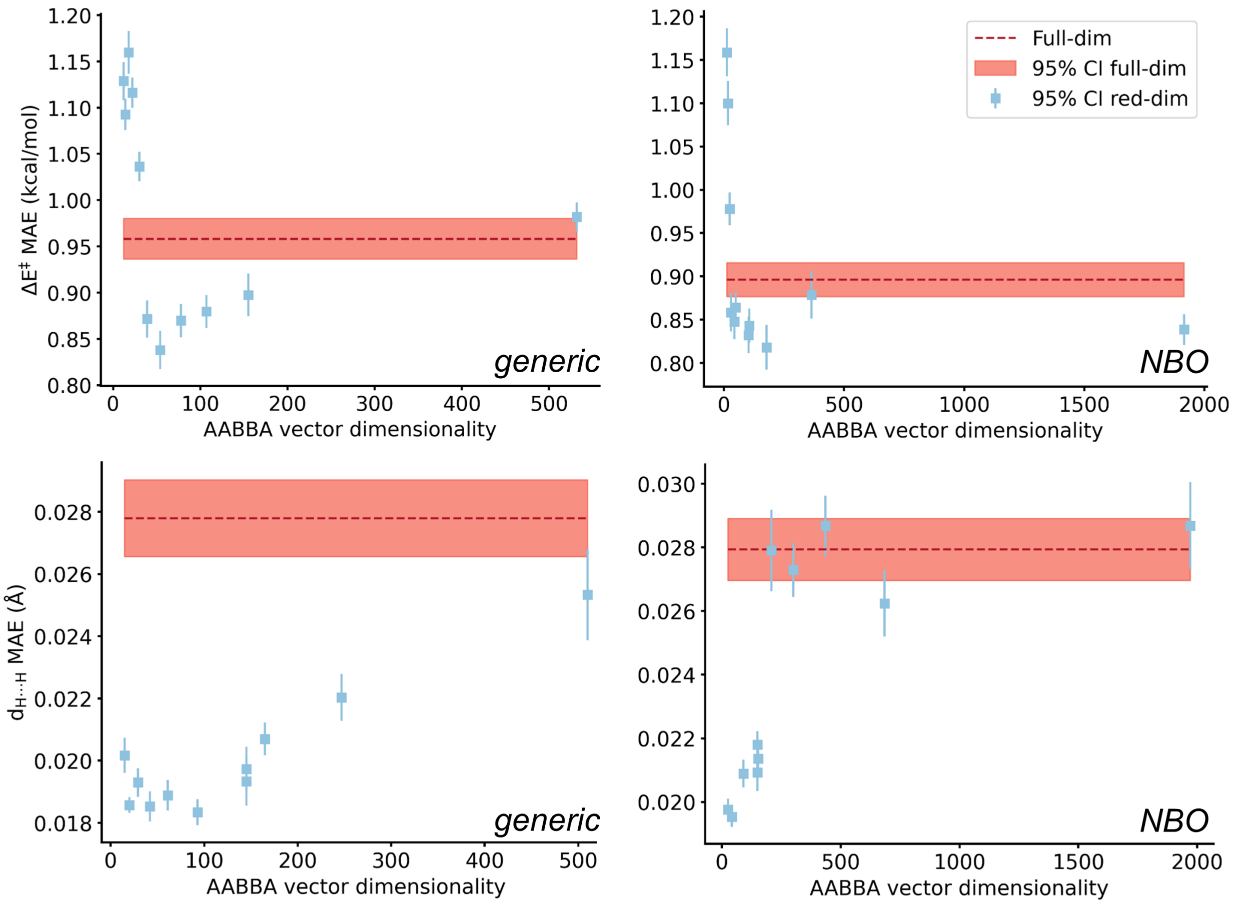

Figure S9: Influence of reducing the dimensionality of the AABBA representation in the MAEs of the NNs predicting the Vaska's barriers (ΔE‡; top) and distances (d$_{H \cdots H}$; bottom) from generic (left) and NBO (right) properties. The legend applies to all four plots; CI = confidence interval.

Due to the similarity in the performance of the GP and NN models relative to the dimensionality reduction of the input, we used the optimal dimensions found with the higher resolution GP data for building the NN models.

References

(1) Moreau, G.; Broto, P. Autocorrelation of a topological structure: A new molecular descriptor. *Nouv. J. Chim.* **1980**, *4*, 359–360.

(2) Janet, J. P.; Kulik, H. J. Resolving Transition Metal Chemical Space: Feature Selection for Machine Learning and Structure–Property Relationships. *J. Phys. Chem. A* **2017**, *121*, 8939–8954.

(3) Friederich, P.; dos Passos Gomes, G.; De Bin, R.; Aspuru-Guzik, A.; Balcells, D. Machine Learning Dihydrogen Activation in the Chemical Space Surrounding Vaska's complex. *Chem. Sci.* **2020**, *11*, 4584–4601.

(4) Kneiding, H.; Lukin, R.; Lang, L.; Reine, S.; Pedersen, T. B.; de Bin, R.; Balcells, D. Deep Learning Metal Complex Properties with Natural Quantum Graphs. *Digital Discovery* **2023**, *2*, 618–633.

(5) Paszke, A. et al. *Advances in Neural Information Processing Systems 32*; Curran Associates, Inc., 2019; pp 8024–8035.

(6) Friedman, J. H. Greedy Function Approximation: A Gradient Boosting Machine. *The Annals of Statistics* **2001**, *29*, 1189 – 1232.

(7) Friedman, J. H. Stochastic Gradient Boosting. *Computational Statistics & Data Analysis* **2002**, *38*, 367–378.

(8) Pedregosa, F. et al. Scikit-learn: Machine Learning in Python. *Journal of Machine Learning Research* **2011**, *12*, 2825–2830.

(9) Gardner, J. R.; Pleiss, G.; Bindel, D.; Weinberger, K. Q.; Wilson, A. G. GPyTorch: Blackbox Matrix-Matrix Gaussian Process Inference with GPU Acceleration. Advances in Neural Information Processing Systems. 2018.
